# Recent progress in quantitative analysis of self‐assembled peptides

**DOI:** 10.1002/EXP.20230064

**Published:** 2024-01-23

**Authors:** Xiaoyao Cai, Wei Xu, Chunhua Ren, Liping Zhang, Congrou Zhang, Jianfeng Liu, Cuihong Yang

**Affiliations:** ^1^ Key Laboratory of Radiopharmacokinetics for Innovative Drugs, Chinese Academy of Medical Sciences, Tianjin Key Laboratory of Radiation Medicine and Molecular Nuclear Medicine, Institute of Radiation Medicine Chinese Academy of Medical Sciences & Peking Union Medical College Tianjin P. R. China; ^2^ Department of Pathology Characteristic Medical Center of Chinese People's Armed Police Forces Tianjin P. R. China; ^3^ Metabolomics and Analytics Center, Leiden Academic Centre of Drug Research Leiden University Leiden The Netherlands

**Keywords:** Imaging techniques, LC‐MS, Molecular dynamics simulation, Quantitative analysis, Self‐assembled peptides

## Abstract

Self‐assembled peptides have been among the important biomaterials due to its excellent biocompatibility and diverse functions. Over the past decades, substantial progress and breakthroughs have been made in designing self‐assembled peptides with multifaceted biomedical applications. The techniques for quantitative analysis, including imaging‐based quantitative techniques, chromatographic technique and computational approach (molecular dynamics simulation), are becoming powerful tools for exploring the structure, properties, biomedical applications, and even supramolecular assembly processes of self‐assembled peptides. However, a comprehensive review concerning these quantitative techniques remains scarce. In this review, recent progress in techniques for quantitative investigation of biostability, cellular uptake, biodistribution, self‐assembly behaviors of self‐assembled peptide etc., are summarized. Specific applications and roles of these techniques are highlighted in detail. Finally, challenges and outlook in this field are concluded. It is believed that this review will provide technical guidance for researchers in the field of peptide‐based materials and pharmaceuticals, and facilitate related research for newcomers in this field.

## Graphical abstract

Self‐assembled peptides have been among the important biomaterials due to its excellent biocompatibility and diverse functions. This review summarizes the techniques for quantitative analysis, including imaging‐based quantitative techniques, chromatographic technique, and computational approach. These techniques can facilitate the investigation of the structure, properties, biomedical applications, and even supramolecular assembly processes of self‐assembled peptides. The perspective and outlooks of quantitative‐analysis techniques for self‐assembled peptides are also given in detail.

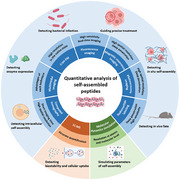

## 1 INTRODUCTION

In nature, biological molecules, such as peptides, phosphatides, and nucleic acids can self‐assemble into supramolecular structures in a bottom‐up manner. Self‐assembly is a ubiquitous phenomenon which involves multi‐scale events from atomic level to molecular level, including intermolecular interactions, orderly folding and stacking, interactions between molecules and their surroundings etc.^[^ ^1^, ^2^ ^]^ Since a repeated peptide sequence (EAK16) isolated from yeast protein was first discovered to form nanofibrous hydrogel in 1990s,^[^ ^3^ ^]^ self‐assembled peptides have been extensively and intensively investigated. 20 different natural amino acids are harnessed to synthesize peptides, generating a wide range of peptide sequences. Peptides with specific sequences and properties can spontaneously self‐assemble into various ordered nanostructures in aqueous solution, including nanofiber,^[^ ^4^, ^5^, ^6^ ^]^ nanotube,^[^ ^7^, ^8^ ^]^ nanosphere,^[^ ^9^, ^10^ ^]^ nanosheet,^[^ ^11^ ^]^ and vesicle.^[^ ^12^, ^13^ ^]^ The driving forces are mainly non‐covalent interactions, such as hydrogen bond, hydrophobic interaction, π─π interaction, and electrostatic interaction.^[^ ^14^, ^15^ ^]^ For example, it is found that natural self‐assembled peptides containing di‐phenylalanine exhibited excellent self‐assembled ability, due to the π─π stacking of side chain benzene rings.^[^ ^16^ ^]^ The in vitro formation of self‐assemblies can be achieve by heating‐cooling,^[^ ^17^ ^]^ enzyme catalysis,^[^ ^18^ ^]^ metal coordination^[^ ^19^ ^]^ etc. Besides, in situ self‐assembly, namely formation of self‐assemblies at the target site in vivo, can be accomplished via the response to microenvironment such as specific receptor on the cell membrane,^[^ ^4^ ^]^ overexpressed enzyme,^[^ ^20^ ^]^ and acidic environment.^[^ ^21^ ^]^ More importantly, the functionalities of self‐assembled peptides can also be precisely and modularly designed by introduction of various functional components, such as responsive sequences,^[^ ^22^, ^23^, ^24^ ^]^ targeting motifs,^[^ ^25^, ^26^, ^27^ ^]^ and therapeutic agents.^[^ ^28^ ^]^ Benefiting from delicate nanostructures and varied functional modules, self‐assembled peptides present improved biostability,^[^ ^29^ ^]^ long circulation in vivo,^[^ ^30^ ^]^ and more powerful and diverse biological functionalities.^[^ ^31^ ^]^ Therefore, multi‐functional self‐assembled peptides have been widely applied to diagnosis^[^ ^32^, ^33^, ^34^ ^]^ and therapy^[^ ^35^, ^36^, ^37^ ^]^ of diseases.

**FIGURE 1:**
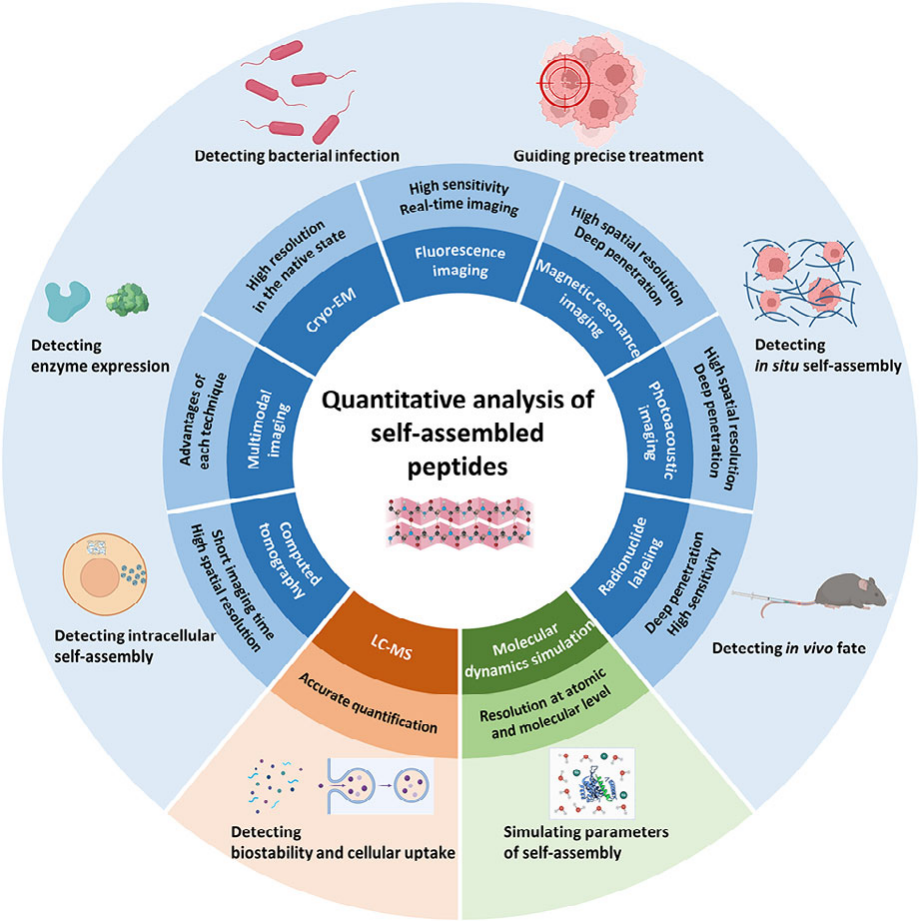
Schematic illustration of techniques for quantitative analysis of self‐assembled peptides.

With the deepening understanding of self‐assembled peptides over the decades, it is found that quantitative analysis of concentration, existing state and behaviors of self‐assembled peptides plays an instructive role in investigating their biological properties^[^ ^38^ ^]^ and performing functions.^[^ ^39^ ^]^ For example, determination of the in situ concentration of self‐assembled peptides can provide guidance for precise therapy, achieving a better therapeutic gain.^[^ ^28^, ^40^ ^]^ Moreover, quantitatively deciphering complex processes of peptide self‐assembly at atomic and molecular level assists in revealing the relationship between molecular structure and self‐assembly process as well as morphology of the assemblies.^[^ ^41^, ^42^ ^]^ To this end, a wide range of techniques have been adopted, including fluorescence imaging (FLI),^[^ ^38^, ^43^ ^]^ magnetic resonance imaging (MRI),^[^ ^40^, ^44^ ^]^ photoacoustic imaging (PAI),^[^ ^28^, ^45^ ^]^ radionuclide labeling (RL),^[^ ^29^, ^46^ ^]^ computed tomography (CT),^[^ ^47^, ^48^ ^]^ multimodal imaging,^[^ ^49^ ^]^ cryo‐electron microscopy,^[^ ^50^, ^51^, ^52^ ^]^ liquid chromatography‐mass spectrometry (LC‐MS),^[^ ^18^, ^53^ ^]^ and computational molecular dynamics (MD) simulation^[^ ^41^, ^54^ ^]^ (Figure 1). Each of these techniques has unique strengths and applications. For imaging‐based techniques, quantifying signal intensity of self‐assembled peptides enables various applications. For example, RL with deep‐penetration ability is invariably applied to the study in vivo biodistribution of self‐assembled peptides,^[^ ^55^ ^]^ while highly sensitive FLI is suitable for the detection of cellular uptake.^[^ ^38^ ^]^ Conventional MRI and emerging PAI with excellent spatial resolution have been exploited to quantify the concentration of in situ formed nanomaterials and calculate aggregation degree in vivo.^[^ ^28^, ^40^, ^44^ ^]^ In contrast with semi‐quantitation imaging‐based techniques, LC‐MS comes as a potent tool for accurate quantification of in vitro, intracellular and ex vivo samples, particularly for analyzing the stability of peptides to various exposures.^[^ ^29^, ^53^ ^]^ Additionally, the emerging computational MD simulation serves to calculate assembly‐related parameters, probe molecular conformation, quantify intermolecular interactions etc., which advances our understanding of mechanism of peptide self‐assembly in an unprecedented way.^[^ ^56^, ^57^ ^]^ Therefore, the proper application of these tools will contribute to the work of researchers in the whole process from peptide molecular design to their biomedical applications.

Up to date, reviews concerning nanostructures, designing rationale and biomedical application of self‐assembled peptides have been widely reported.^[^ ^58^, ^59^ ^]^ However, there are few comprehensive reviews focused on the techniques for quantitative analysis. Herein, we review the recent studies which utilize quantitative‐analysis tools to investigate properties and functions of self‐assembled peptides, mechanism of supramolecular self‐assembly etc. The relevant techniques and their advantages are summarized, and specific applications are divided in detail (Table 1). Subsequently, the roles of quantitative analysis are specifically described. Finally, the conclusion and outlooks of quantitative‐analysis techniques for self‐assembled peptides are given. We believe that this review will aid researchers in further understanding the importance of quantitative‐analysis techniques in the performance characterization and biomedical applications of self‐assembled peptides in vivo and in vitro, which contributes to designing more functional and powerful peptide‐based nanomaterials.

**TABLE 1:** Techniques for quantitative analysis of self‐assembled peptides.

	Techniques	Advantages	Applications	Refs.
Imaging‐based techniques	Fluorescence imaging	High sensitivity, real‐time imaging	1. Detecting biomarker	[39, 43, 66]
			2. Detecting in situ self‐assembly	[38, 70, 73]
	Magnetic resonance imaging	High spatial resolution, deep penetration	1. Guiding precise treatment	[40, 81]
			2. Detecting the enzyme expression	[83, 84, 85]
			3. Detecting aggregation degree	[44]
	Photoacoustic imaging	High spatial resolution, deep penetration	1. Guiding precise therapy	[28, 97]
			2. Detecting chemotaxis of macrophage	[99]
			3. Detecting in situ formation of assemblies	[45, 101]
	Radionuclide labeling	Deep penetration, high sensitivity	Detecting in vivo fate	[29, 46, 106]
	Computed tomography	Short imaging time, high spatial resolution	Detecting intracellular self‐assembly	[47, 48]
	Multimodal imaging	Integrated advantages of each technique	Detecting biomarker	[112, 113]
	Cryo‐EM	High resolution in the native state	Describing nanostructures	[51, 52]
Chromatographic technique	LC‐MS	Accurate quantification	Detecting biostability and cellular uptake	[18, 29, 53]
Computational technique	Molecular dynamics simulation	Fast simulation at atomic and molecular level	Simulating parameters of self‐assembly	[33, 41, 42, 131]

## 2 IMAGING‐BASED TECHNIQUES FOR QUANTITATIVE ANALYSIS OF SELF‐ASSEMBLED PEPTIDES

### 2.1 Fluorescence imaging

Fluorescence imaging (FLI) constitutes a cost‐effective visualization tool that detect photons emitted from fluorescent probes. FLI can produce highly sensitive images for real‐time and quantitative detection of physiological activities and molecular interactions in vivo.^[^ ^60^ ^]^ Generally speaking, the intensity of fluorescence signals are proportional to the concentration of fluorophores, which can be applied to quantitative analysis. Fluorophore‐conjugated self‐assembled peptides are usually applied to in vivo superficial detection or in vitro study,^[^ ^61^, ^62^ ^]^ due to the limitation of tissue penetration. However, since physiological environments are filled with water, these traditional fluorophores (e.g. rhodamine and Cy 5) are preferred to aggregate in most application scenarios, leading to quenched fluorescence, which compromises the accuracy of qualitative and quantitative analysis of self‐assembled peptide. Conversely, a class of fluorescent molecules with aggregation‐induced emission (AIE) effect show enhanced fluorescence in the aggregation state,^[^ ^63^ ^]^ rendering FLI more practical for biological applications. In addition to AIEgens, environmentally sensitive fluorophore NBD is also found to yield enhanced fluorescence in hydrophobic environment.^[^ ^64^ ^]^ Therefore, NBD and AIEgens are ideal indicator for sensing peptide self‐assembly.

#### 2.1.1 FLI for quantitatively detecting biomarkers

According to previous studies, AIEgen‐conjugated self‐assembled peptide could produce the enhanced fluorescence emission under certain conditions, such as the enzyme catalysis and ligand‐receptor interaction. For enzyme catalysis, AIEgen‐peptide conjugates with stimuli‐responsive peptide sequences would light up fluorescence in certain microenvironment, serving to indicate pathological biomarkers.^[^ ^65^ ^]^ For example, Ding's group established a highly sensitive AIE fluorescent probe, TPE‐GFFYK (DVEDEE‐Ac), to detect the activity of protease caspase‐3, which is a major mediator of apoptosis in some diseases.^[^ ^43^ ^]^ After peptide sequence (DEVD) was cleaved by caspase‐3, the residues self‐assembled into nanofibers due to the change of hydrophilicity, turning on fluorescence. At the same concentration, the fluorescence intensity of TPE‐GFFYK residues after enzymolysis was much higher than that of the control group (without self‐assembly). Remarkably, the detection limit of TPE‐GFFYK (DVEDEE‐Ac) was calculated to be 0.54 pm, which was much lower than that of unassembled TPE‐K(DEVD‐Ac) (3.50 pm), indicative of an enhanced sensitivity by self‐assembly. In addition, it was found that the probe had excellent selectivity towards caspase‐3 over enzymes (e.g. pepsin, trypsin, lysosome etc.). The above results demonstrate that the brightness of conjugated AIEgen can directly and quantitatively reflect the assembly state of supramolecular peptides and indirectly detect the activity of enzymes and other markers. Additionally, Zhang et al. designed a fluorescent alkaline phosphatase‐sensitive probe based on self‐assembled peptide. The probe could self‐assemble into nanoaggregate triggered by ALP‐catalyzed, switching on fluorescence. It exhibited excellent selectivity and sensitivity towards ALP, lowering the detection limit to 6.6 × 10^−3^ U mL^−1^.^[^ ^66^ ^]^

**FIGURE 2:**
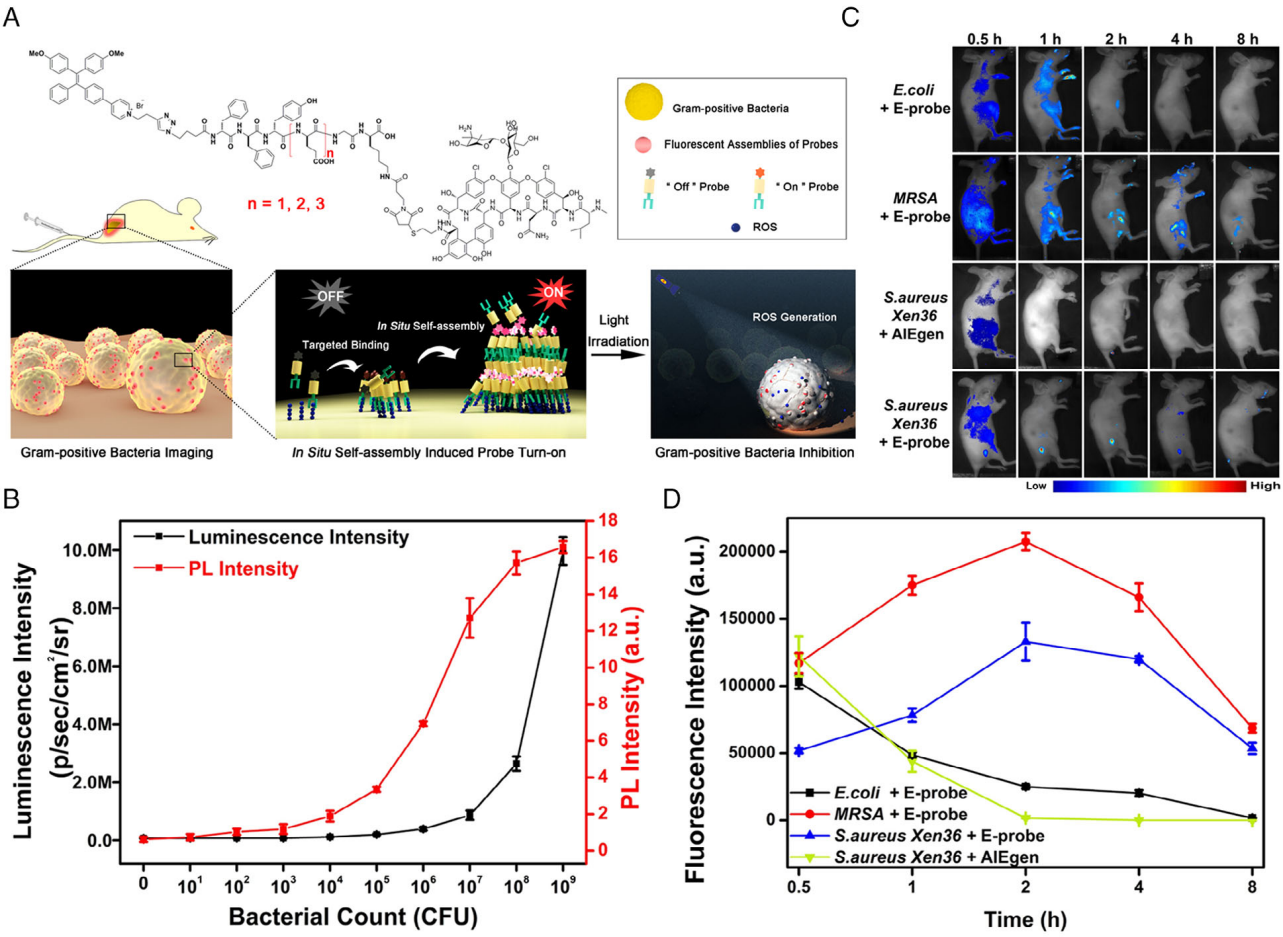
Fluorescence imaging for the detection of bacterial infections using self‐assembly of peptide‐based probe. (A) E‐probe molecular structure and schematic of diagnosis and treatment of Gram‐positive bacterial infections by in situ self‐assembly of peptide‐based AIEgen probe. (B) Detection sensitivity of E‐probe compared with bioluminescence. (C) Fluorescent images of bacteria‐bearing mice treated with E‐probe or AIEgen at different time point. (D) Quantification of fluorescence intensity of infected sites in different groups. Reproduced with permission.^[^ ^39^ ^]^ Copyright, 2020 Elsevier.

Besides, ligand‐receptor interactions can elicit the assembly of AIEgen‐peptide conjugates as well, which can be used to detect the bacterial infection in vivo. Yang et al. constructed a theranostic probe (E‐probe) based on self‐assembled peptide and AIEgen (TPE) (Figure 2A). Antibiotic vancomycin was conjugated to the C‐terminus for targeting to Gram‐positive bacteria. Due to the intrinsic capability of phenylalanine‐phenylalanine (FF), binding to the surface of bacteria would result in self‐assembly of the probe, generating fluorescence. Bioluminescent bacterial strain, multidrug resistant *S. aureus* Xen36, was employed to evaluate the sensitivity of E‐probe. In terms of bioluminescence emitted from *S. aureus* Xen36, the detection window ranged from 10^6^ to 10^9^ CFU mL^−1^; in terms of fluorescence of E‐probe, the detection window ranged from 10^3^ to 10^9^ CFU mL^−1^, decreasing the detection limit by nearly 1000 folds (Figure 2B). Subsequentially, in vivo infection imaging and quantitative analysis (Figure 2C,D) illustrated that E‐probe had selectivity towards Gram‐positive bacteria over Gram‐negative bacteria. Moreover, obvious different fluorescence intensity could be observed between *S. aureus* Xen36 and MRSA. Considering that *S. aureus* Xen36 is moderately resistant to vancomycin and MRSA is sensitive to vancomycin, the fluorescence intensity may serve as an indicator for level of resistance to bacterial strain. In addition to producing fluorescence, AIEgens also yield photosensitization effect, generating reactive oxygen species (ROS) upon light irradiation. Therefore, the photodynamic therapy, employing light to activate photosensitizer to generate ROS for killing cells, could be initiated based on the quantification of fluorescence intensity, precisely and efficiently killing Gram‐positive bacteria.^[^ ^39^ ^]^

#### 2.1.2 FLI for quantitatively detecting in situ self‐assembly

According to previous studies, pre‐assembled peptides might be eliminated by reticuloendothelial system upon injection, suffering the low bioavailability and undesired side effects.^[^ ^67^ ^]^ On the contrary, in situ self‐assembled peptides can form self‐assemblies at the target sits from molecules triggered by site‐specific or disease‐specific stimulus, which circumvents the reticuloendothelial system.^[^ ^68^, ^69^ ^]^ Accordingly, the in situ self‐assembly has become a promising strategy for promoting delivery efficiency of self‐assembled peptides. Meanwhile, the investigation of in situ self‐assembly behavior at the level of tissue, cell and organelle plays a significant role in construction and optimization of self‐assembled peptides.

**FIGURE 3:**
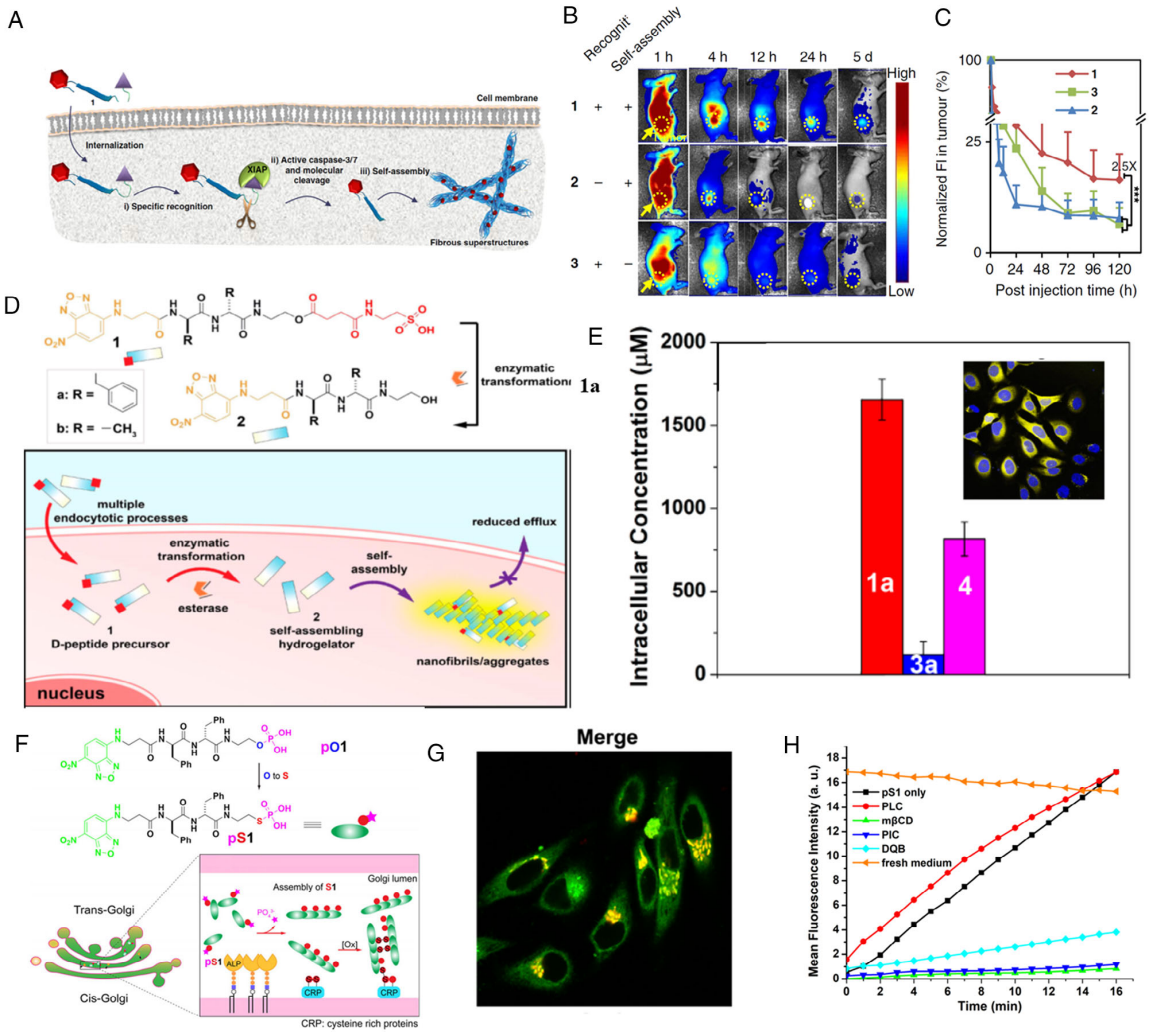
Fluorescence imaging for quantitative detection of peptide in situ self‐assembly. (A) Mechanism of the tumor selective cascade activation self‐detained system (TCASS). (B) Fluorescence images of molecules 1, 2, and 3 on H460 tumor‐bearing mice after injection. (C) Normalized fluorescence intensity in tumor. Reproduced under the terms of CC BY‐NC license.^[^ ^70^ ^]^ Copyright 2019, An et al. (D) Molecular structure of precursor 1a and schematic of taurine enhancing cellular uptake of D‐peptide and self‐assembly into nanofiber. (E) Intracellular concentration of 1a in HeLa cells after incubation for 24 h. CLSM images of HeLa cells treated with 1a for 24 h (Top right). Reproduced under an ACS AuthorChoice License.^[^ ^38^ ^]^ Copyright 2015, Zhou et al. (F) Molecule structure of thiophosphopeptide and mechanism of instantly targeting the Golgi apparatus by enzymatic assembling and forming disulfide bonds. (G) CLSM images of HeLa cells treated with pS1 for 8 min and stained with CellLight Golgi‐RFP. (H) Time‐dependent mean fluorescence intensity of Golgi in HeLa cells pretreated with different inhibitors. Reproduced under the terms of CC BY‐NC license.^[^ ^73^ ^]^ Copyright 2021, Tan et al.

In the study of Zhao's group, they investigated the retention of self‐assembled peptides in the tumor tissue by quantifying the fluorescence.^[^ ^70^ ^]^ A tumor selective cascade activation self‐detained system (TCASS) was constructed, which could recognize the X‐linked inhibitor of apoptosis protein (XIAP) (Figure 3A). After the recognition, downstream caspase‐3/7 were activated to cleave the enzymatically responsive linker, triggering the in situ self‐assembly, after which the fluorescence of NBD was activated. The quantitative fluorescence in vivo results (Figure 3B,C) show that, in situ self‐assembled molecule displayed approximately 22.0% accumulation efficiency 48 h after intravenous administration, which is higher than that of control molecules. More importantly, according to fluorescence intensity, in situ self‐assembled molecule remained above 16.0% on the fifth day, nearly 2.5 times as high as the value of control molecules.

Additionally, Xu and coworkers quantitatively detected the intracellular fluorescence of self‐assembly, aiming at investigating the cellular uptake of taurine‐modified peptide. Since the superb biostability of d‐peptides is proved,^[^ ^29^ ^]^ d‐peptides have been widely applied in construction of nanomaterials.^[^ ^71^ ^]^ However, compared with l‐counterparts, some d‐peptides exhibit low endocytosis resulting from its inadequate interaction with endogenous transporter.^[^ ^72^ ^]^ Xu's group introduced natural taurine into d‐peptide to significantly enhanced the cellular uptake.^[^ ^38^ ^]^ In the meantime, NBD moiety was conjugated to the N‐terminus of the peptide, in order to indicate the formation of self‐assembly. As shown in Figure 3D, the precursor molecule (1a) consisted of NBD, self‐assembling sequence (d‐Phe‐d‐Phe), enzyme cleavage site (ester bond), and a taurine residue. Upon taken up by the cells, the taurine group of 1a was removed by enzyme, eliciting self‐assembly of the residue (2a) into nanofibers and generating fluorescent signals of NBD. The molecule (3a) without taurine residue acted as control. As shown in Figure 3E, after incubation with HeLa cells, significant fluorescence emitted from cells treated with 1a could be observed. Quantification of fluorescence indicated that taurine boosted endocytosis of d‐peptide by more than 10 times.

Besides, Xu and coworkers also designed a Golgi‐targeting NBD‐bearing thiophosphopeptide (pS1) (Figure 3F).^[^ ^73^ ^]^ The mechanism of endocytosis and Golgi accumulation induced by enzymatic dephosphorylation was studied by examining the rate of fluorescence increase in Golgi. Upon using different inhibitors of endocytosis, the fluorescence quantification of Golgi (Figure 3G,H) indicated that pS1 enter cells mainly by caveolin‐mediated endocytosis and micropinocytosis. Moreover, using different inhibitors of phosphatases resulted in different accumulating rate of pS1 at the Golgi, suggesting that in addition to the ALP, other phosphatases also engaged in the enzymatic accumulation in Golgi.

### 2.2 Magnetic resonance imaging

Magnetic resonance imaging (MRI) has been among the modern diagnostic technologies commonly used in clinic, due to its unique advantages of noninvasiveness, superb spatial resolution, and excellent tissue penetration.^[^ ^74^ ^]^ Excellent penetration ability of MRI allows for imaging, detection and investigation in deep tissues and organs. However, MRI often suffers low imaging resolution, because the differences in relaxation time between normal tissue and tumor are small. Contrast agents (CAs) can reduce longitudinal relaxation (*T*_1_) or transverse relaxation time (*T*_2_) by affecting the relaxation of surrounding water protons, improving the resolution of MRI.^[^ ^75^ ^]^ Meanwhile, the relaxation rate (1/*T*_1_ of 1/*T*_2_) is directly proportional to the CAs concentration. Therefore, based on the relaxation rate measured at the known CAs concentration, the unknown concentration can be calculated correspondingly. To date, a wide range of paramagnetic metal contrast agents has been developed and applied to boost the imaging efficacy in clinic, including gadolinium (Gd^3+^), manganese (Mn), and iron oxide nanoparticles (Fe_3_O_4_).^[^ ^76^, ^77^, ^78^ ^]^ Meanwhile, self‐assembled peptides are harnessed to coordinate with metal ions to generate novel activable smart contrast agents. The contrast agents‐bearing self‐assembled peptides will be converted into paramagnetic nanostructures in the response to certain stimuli (e.g. enzyme or bacteria), decreasing the T_1_ or T_2_ and enhancing MRI signals.^[^ ^79^ ^]^ Meanwhile, through quantifying the T_1_ or T_2_ and calculating relaxation rate, content of self‐assembled peptide in the tumor can be determined, facilitating the detection of enzyme, bacteria or other biomarkers of interest.

#### 2.2.1 MRI for concentration‐guided in vivo precise treatment

**FIGURE 4:**
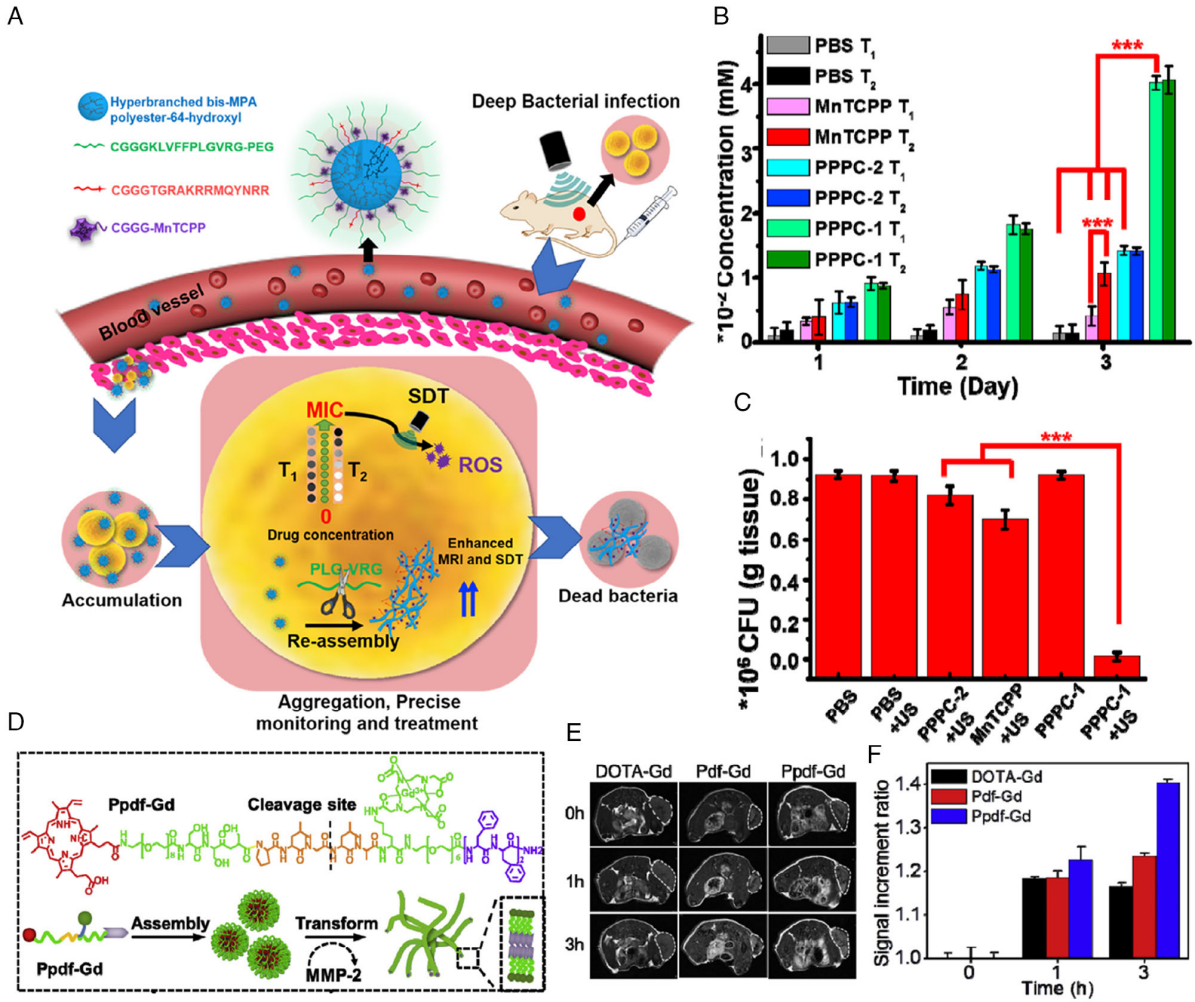
Magnetic resonance imaging for guiding precise treatment in vivo. (A) Schematic of enzyme‐instructed transformation of PPPC for precise MRI‐guided therapy of bacterial infections. (B) Concentration of materials at infected sites quantified by the value of *T*_1_ and *T*_2_. (C) Antibacterial efficacy of different materials. Reproduced with permission.^[^ ^40^ ^]^ Copyright 2021 Elsevier. (D) Illustration of morphological transformation of Ppdf‐Gd from spherical nanoparticles to nanofibers triggered by MMP‐2. (E) T1‐weighted MRI images of mice at 0, 1, and 3 h after intravenous injection. (F) Signal increment ratio in tumor area. Reproduced with permission.^[^ ^81^ ^]^ Copyright 2018 Elsevier.

Precise treatment has attracted substantial attention amongst scientific researchers and clinicians. MRI possesses the advantage of deep penetration, which is suitable for indicating the pathological changes and guiding precise treatment for serious diseases, such as bacterial infections and tumors. Recently, Wang's group developed a polymer‐peptide‐porphyrin conjugate (PPPC) to precisely combat drug‐resistant deep infection using sonodynamic therapy (SDT) (Figure 4A).^[^ ^40^ ^]^ SDT is an emerging therapy which use ultrasound to activate sonosensitizers to produce ROS for killing cancer cells.^[^ ^80^ ^]^ PPPC is consisted of a hyperbranched polymer backbone, a bacteria‐targeting motif, a self‐assembled peptide with an enzyme‐responsive sequence, and an Mn‐containing sonosensitizer. PPPC formed PEG‐shell nanoparticles in aqueous solution, enabling long‐circulation. As the nanoparticles reached the infection sites, the PEG shell would be decomposed by gelatinase, after which PPPC responsively self‐assembled into nano aggregates, rendering more interaction with bacteria and retention of sonosensitizers. The enhanced accumulation significantly decreased the minimal inhibitory concentration (MIC). Of note, as shown in Figure 4B, since the values of r_1_ (1/*T*_1_) and r_2_ (1/*T*_2_) are inextricably correlated to the concentration of Mn, the amount of PPPC at the infection site can be accurately calculated. As the concentration exceeded MIC, SDT was initiated, achieving precise and efficient eradication of bacteria. Figure 4C showed that MRI‐guided SDT (PPPC‐1 together with ultrasound) exerted the best killing efficacy. Therefore, thanks to the guidance of MRI, the precise SDT possesses deep tissue penetration, real‐time concentration monitoring, and enhanced therapeutic efficacy on drug‐resistant bacterial infection.

Apart from the treatment of bacterial infections, MRI can also be utilized in the therapy for tumors. Zhang and coworkers fabricated an matrix metalloproteinase‐2 (MMP‐2) responsive chimeric peptide for MRI and precise PDT (Figure 4D).^[^ ^81^ ^]^ Under the physiological condition, the chimeric peptide self‐assembled into nanospheres, and in the MMP‐2 overexpressed tumor area, it would be cleaved by MMP‐2 and converted from nanospheres to nanofibers, which concentrated the contrast agents and increased the value of *r*_1_ (1/*T*_1_). More importantly, the changes in the value of *r*_1_ was monitored to determine the content of photosensitizer in the tumor (Figure 4e,f), guiding the PDT more precisely.

#### 2.2.2 MRI for quantitatively detecting enzyme expression

The abnormal expression or overexpression of enzymes in tumor tissues is invariably linked to the occurrence, progression and deterioration of illness.^[^ ^82^ ^]^ Thus, determination of enzyme expression is vital for diagnosis and treatment of tumors. By introducing enzyme‐responsive peptide sequences into CAs‐bearing self‐assembled peptides, the formation of CAs self‐assemblies could be achieved via the response to enzymatic catalysis, which could concentrate CAs and generate intense MRI signals. The intensity of MRI signals is highly related to the expression level of enzyme. Accordingly, the enzyme activity can be, in turn, determined by detecting the relaxation time (*T*_1_ or *T*_2_) and calculating relaxation rate (r_1_ or r_2_) of CAs. Hua et al. constructed a phosphorylated tyrosine‐containing peptide‐Ga^3+^ complex (Nap‐GFFpYGRGD‐Ga^3+^) that could be converted into hydrogel by alkaline phosphatase (ALP), quantifying the enzyme activity in different cell lines by MRI signals. Since ALP is related to some diseases including tumor and bacterial infection, the designed Gd‐peptide hydrogel could be used to quantitatively test ALP activity and diagnose diseases.^[^ ^83^ ^]^ Similarly, Dong et al. introduced a commercial metal‐coordination agent (DOTA) and constructed a Gd‐peptide conjugated precursor (Nap‐FFFpY‐EDA‐DOTA(Gd)) which can yield supramolecular hydrogel through dephosphorylation.^[^ ^84^ ^]^ The *r*_2_ (relaxation rate, 1/*T*_2_) of Gd‐containing hydrogel (61.5 mm ^−1^S^−1^) was distinctly higher than that of DOTA‐Gd (4.40 mm ^−1^S^−1^), indicating that a much greater contrast enhancement and stronger signals were attained. In vivo MR images corroborated that ALP enhanced the *T*_2_ weighted signal, which could be harnessed to determine the enzyme activity. Accordingly, the designed Gd‐peptide hydrogel hold promise in quantitative detection of ALP activity and diagnose diseases.

Liang's group fabricated a Caspase‐3‐activatable self‐assembled probe (DEVDCS‐Gd‐CBT) for apoptosis imaging in vivo.^[^ ^85^ ^]^ Upon cleavage by Caspase‐3 in apoptotic cells, DEVDCS‐Gd‐CBT form a cyclic dimer by the CBT‐Cys click reaction, after which it self‐assembled into Gd nanoparticles, boosting *T*_1_‐weighted MRI in vivo. Accordingly, by quantifying the change of relaxation rate, the DEVDCS‐Gd‐CBT could be used to indicate the Caspase 3 expression and apoptosis‐related diseases.

#### 2.2.3 MRI for quantitatively detecting aggregation degree

**FIGURE 5:**
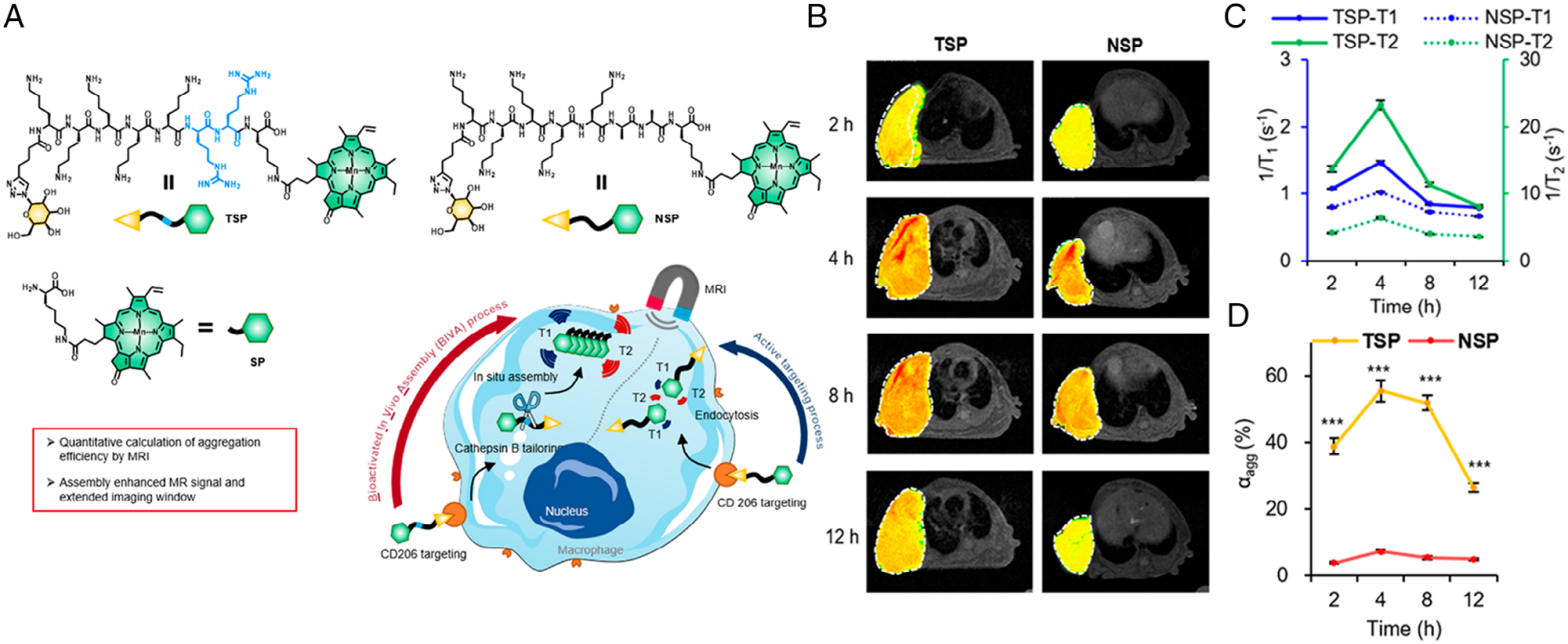
Magnetic resonance imaging for the detection of in vivo aggregation degree of peptide‐based probe. (A) Molecular structure of TSP and NSP and schematic of self‐assembly‐enhanced MRI signals. (B) *T*_1_‐weighted images of tumor‐bearing mice at different time points after intravenous injection of TSP and NSP. (C) The values of 1/*T*_1_ and 1/*T*_2_ at the tumor site of TSP and NSP. (D) Aggregation degree of TSP and NSP at the tumor site calculated by MRI. Reproduced with permission.^[^ ^44^ ^]^ Copyright 2022 American Chemical Society.

Aggregation degree (*α*_agg_), which refers to ratio of assembly to monomer, is an important parameter for evaluating the in vivo self‐assembly efficiency.^[^ ^86^ ^]^ Since nanocarriers are easily cleared by the reticuloendothelial system in vivo, self‐assembled peptides are developed to administrate in the form of small molecules and responsively form nanostructures in situ, benefiting more retention and accumulation at targeted site.^[^ ^87^, ^88^ ^]^ Therefore, determination of *α*_agg_ provides insights into in vivo self‐assembly behaviors. Moreover, it can explicate the alterations of physicochemical properties and biological functions before and after assembly.^[^ ^89^ ^]^ To this end, Luo et al. established peptide‐based MRI probe for quantitative calculation of α_agg_.^[^ ^44^ ^]^ As shown in Figure 5A, the probe (TSP) consisted of three components: (I) mannose that targets the receptors (CD206) of macrophages; (II) hydrophilic sequence KKKKRRK that can be specifically tailored by cathepsin B; (III) Mn‐pheophorbide α (Ppa[Mn]) which can self‐assemble to enhance *T*_1_ signal. TSP actively targeted macrophages via mannose, triggering receptor‐mediated endocytosis. After cleavage by intracellular high‐expression cathepsin B, the residues assembled into nanofibers, enhancing T_1_‐weighted MR signals and separating the signals of monomer and assembly. As shown in Figure 5B–D, *α*_agg_ of TSP in vivo was calculated to reach the highest level of 55.6% at 4 h and keep stable until 8 h, which was much higher than non‐assembled NSP. Therefore, the developed quantitative method and designed MR probe contributes to further investigation of self‐assembly behavior in vivo, fostering the precise design of high aggregation‐degree self‐assembled nanomaterials.

### 2.3 Photoacoustic imaging

Photoacoustic imaging (PAI) is a non‐ionizing emerging imaging method in biomedical field.^[^ ^90^ ^]^ When a pulsed laser is applied to biological tissues, transient thermoelastic expansion of tissue occurs, which can produce ultrasonic signals that will be converted into 2D or 3D images by computers.^[^ ^91^ ^]^ PAI is simultaneously characterized by high spatial resolution, deep tissue penetration and high sensitivity.^[^ ^92^ ^]^ However, only several endogenous biological molecules are applicable to be light absorbers, such as hemoglobin and melanin.^[^ ^93^ ^]^ Therefore, exogenous photoacoustic agents are developed to broaden PAI applications. Subjecting stimulus‐responsive self‐assembly to photoacoustic contrast agent can substantially improve the performance of PAI.^[^ ^94^, ^95^ ^]^ Meanwhile, the intensity of PAI signals are proportional to the content of photoacoustic agents. Accordingly, by quantifying PA signals, the concentration of photoacoustic agents can be calculated, enabling a wide range of purposes, such as precise therapy, biomarker detection, and investigation of in situ self‐assembly.

#### 2.3.1 PAI for concentration‐guided in vivo precisely treatment

**FIGURE 6:**
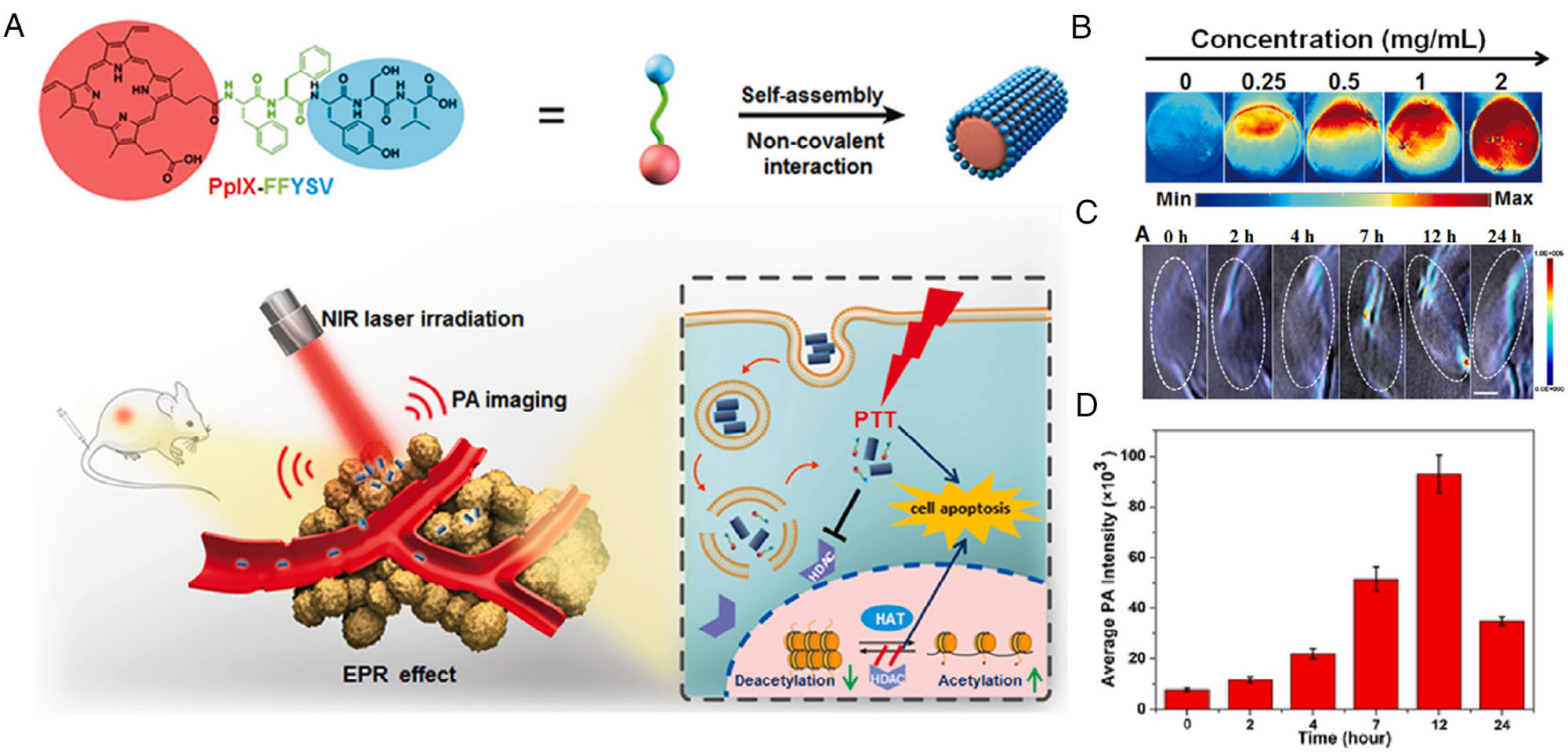
Photoacoustic imaging of self‐assembled peptides for guiding precise treatment in vivo. (A) Molecular structure of PpIX‐FFYSV and schematic of self‐assembly PpIX‐FFYSV for in vivo tumor PAI and guiding combination treatment of chemotherapy and PTT. (B) PAI of different‐concentration PpIX‐FFYSV in PBS. (C) Time‐dependent PA images of tumor after intravenous injection of PpIX‐FFYSV (scale bar = 1 mm). (D Average PAI signals intensity of white dash ellipse area of (C). Reproduced with permission.^[^ ^28^ ^]^ Copyright 2021 Elsevier.

Photoacoustic agents involved in PAI can also generate photothermal effects when applying laser irradiations, which could be employed to photothermal therapy (PTT). Photothermal therapy (PTT) is an emerging treatment which can convert light energy into heat energy to eradicate the cancer cells.^[^ ^96^ ^]^ Liu's group developed a self‐assembled peptide (PpIX‐FFYSV) with photoacoustic imaging function for synergism of PTT and chemotherapy for cancer (Figure 6A).^[^ ^28^ ^]^ PpIX‐FFYSV consisted of photosensitizer porphyrin (PpIX), self‐assembly l‐phenylalanine‐l‐phenylalanine (FF), and anticancer peptide tyroservatide (YSV). PpIX‐FFYSV assembled into nanorod‐like supramolecular structure through heating‐cooling process. PpIX acted as not only a photothermal agent but also a photoacoustic contrast agent. In vitro experiment demonstrated that photoacoustic signals of PpIX‐FFYSV was positively correlated to its concentration (Figure 6B). As shown in Figure 6C,D, through monitoring ultrasonic signals from PpIX in vivo, the supramolecular nanodrug accumulated and reached maximum concentration at the tumor site at 12 h after administration. Subsequently, photothermal therapy was performed precisely under the guidance of PAI. Accordingly, chemo‐photothermal synergistic therapy exerted an approximately 70% in vivo tumor inhibitory rate with the assistance of PAI, significantly enhancing the therapeutic efficacy and decreasing the doses of chemotherapeutic drug.

Similarly, Yan's group synthesized a photothermal peptide‐porphyrin conjugate (TPP‐G‐FF) which could self‐assemble into nanodot in aqueous solution. The photoacoustic signals were observed to reach the highest level at 24 h post injection, providing a guidance of photothermal therapy and yielding an excellent antitumor effect.^[^ ^97^ ^]^

#### 2.3.2 PAI for quantitatively detecting chemotaxis of macrophage in bacterial infections

When tissues are infected by pathogens, macrophages are recruited and gather at the infected site, which can serve as an indicator for early‐stage infection.^[^ ^98^ ^]^ Wang's group developed a peptide‐based photoacoustic agent (MPC) for targeting phagocytic cells in vivo.^[^ ^99^ ^]^ MPC consisted of active‐targeting mannose, caspase‐1 responsive peptide sequence, and photoacoustic agent chlorophyll. It could specifically target macrophages and be internalized considerably. After macrophages were infected by bacteria, caspase‐1 was activated and subsequently cleaved the substrate of MPC, prompting self‐assembly of the residues and producing detectable PA signals. Therefore, chemotaxis of macrophages towards bacterial infection could be tested by quantifying PA signals. Compared with the control groups, a strong PAI signals could be observed in the *S. aureus*‐induced muscular infection, demonstrating the significant chemotaxis to the infection site. The quantitative results indicated that MPC accumulated at the infected site 2.6 times more than in the control site. Since the PA signal was correlated to capsase‐1 tailoring and MPC self‐assembly rather than the number of bacteria, this strategy may offer fairly sensitive early‐stage detection of intracellular infection through quantifying PA signal.

#### 2.3.3 PAI for quantitatively detecting in situ self‐assembly

In situ self‐assembly, such as molecular self‐assembly and nanostructure morphological transformation, has been proved to be potent strategies for prolonging circulation, promoting retention and enhancing delivery efficiency. Meanwhile, introduction of photoacoustic agent into self‐assembled peptides can generate enhanced PAI signals and indicate formation of self‐assemblies, availing in the comprehension of dynamic assembly process. Aggregation degree (*α*_agg_) is an important parameter for evaluating molecular self‐assembling ability.^[^ ^100^ ^]^ Peng and coworkers developed a ratiometric PAI for real‐time quantitative evaluation of aggregation efficiency in vivo.^[^ ^101^ ^]^ The PAI probe (P‐RT, P_18_‐MLGFFQQPKPRVSNKYFSNIHW) was composed of three major components: purpurin 18 (P_18_), peptide sequence responsive to cathepsin E (MLGFFQQPKPR), and peptide ligand (VSNKYFSNIHW) for urokinase plasminogen activator receptor (uPAR). The ratiometric PAI signal (PA_745nm/690 nm_) of P‐RT was calculated to be exponentially related to *α*_agg_. Active targeting and responsive aggregation can promote the accumulation of P‐RT in tumor cells. Quantification results showed that the *α*_agg_ reached the summit of 58% at 2 h in cells and 36% at 6 h in murine xenografted pancreatic tumor model. Therefore, the established ratiometric PAI method enables the real‐time detection of aggregation degree in vivo.

**FIGURE 7:**
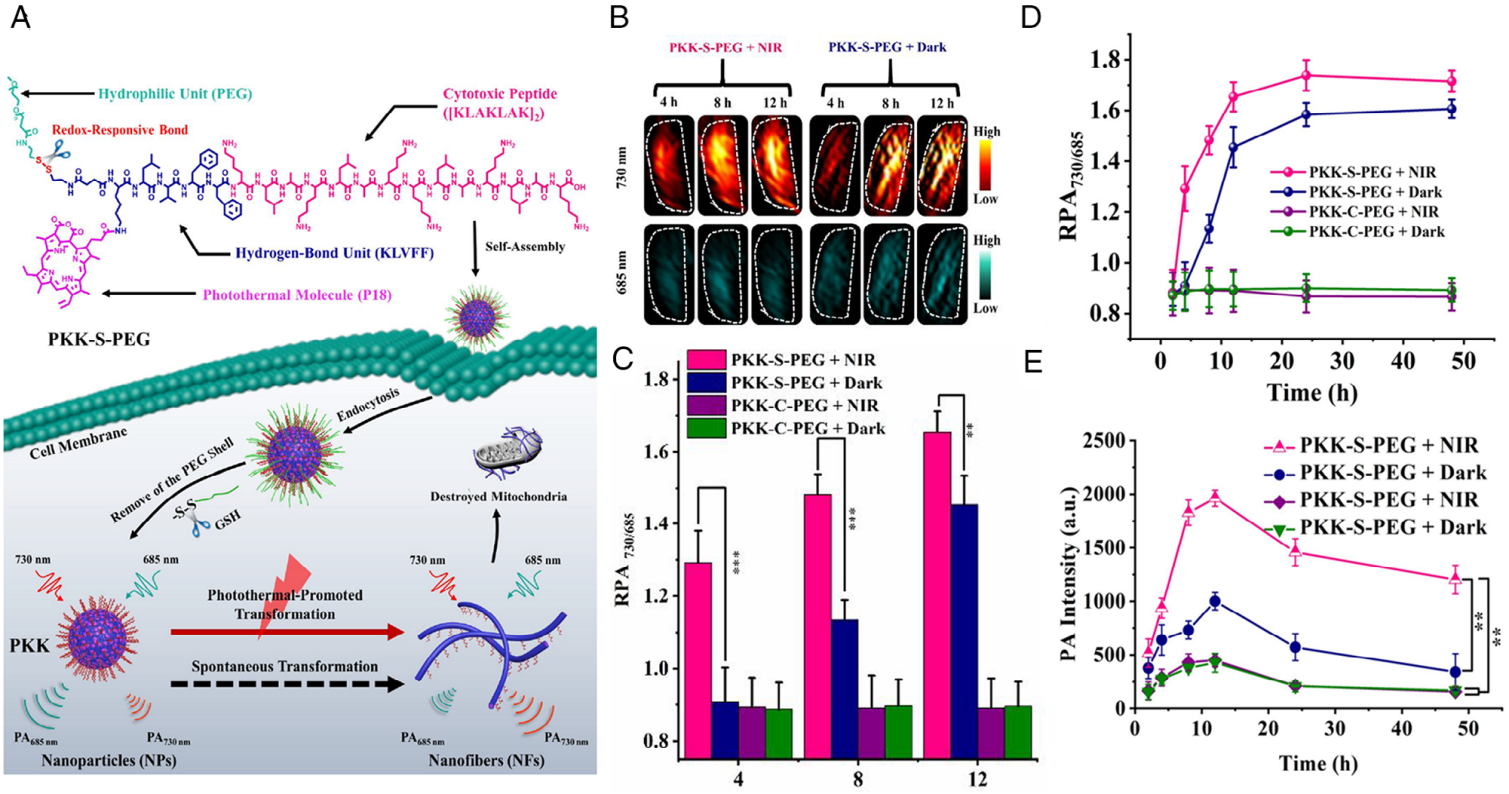
Photoacoustic imaging for quantitatively detecting in situ self‐assembly of peptide derivatives. (A) Molecular structure of PKK‐S‐PEG and schematic of promoted transformation from nanoparticle to nanofiber by photothermal effect, and the process is monitored by PAI. (B)Transverse PA images of tumor at 730 and 685 nm within 4 to 12 h upon injection of PKK‐S‐PEG. (C) Quantification of RPA_730/685_ signals at the tumor site within 4 to 12 h upon injection of PKK‐S‐PEG. (D) Time‐dependent RPA_730/685_ signals from 2 to 48 h after injection of PKK‐S‐PEG. (E) Quantification of PA signal intensity at 730 nm at tumor site from 2 to 48 h after injection of PKK‐S‐PEG. Reproduced with permission.^[^ ^45^ ^]^ Copyright 2020 American Chemical Society.

In addition to molecular self‐assembly, nanostructure morphological transformation from nanoparticles to nanofibers is a common strategy of peptide‐based in situ self‐assembly, which can prolong circulation and increase accumulation.^[^ ^102^ ^]^ Zhang et al. recently constructed a photothermal‐promoted morphology transformation strategy (Figure 7A).^[^ ^45^ ^]^ In this study, a reduction‐responsive polymer‐peptide conjugate (PKK‐S‐PEG), which contained a photothermal molecule purpurin‐18 (P18), was designed. PKK‐S‐PEG could responsively transform from nanoparticles (NPs) in aqueous solution into nanofibers (NFs) after cleaved by GSH in tumor cells, but with slow speed. P18 was introduced to accelerate the transformation process by raising temperature upon laser irradiation. In addition to promoting transformation, the ratiometric photoacoustic images (RPA_730/685_) of P18 at 730 nm/685 nm were harnessed to in situ visually and quantitatively evaluate the rate of transformation from NPs to NFs. Accordingly, in vivo images and quantitation of PAI (Figure 7B–D) indicated that, compare with PKK‐S‐PEG + dark group, photothermal effect could significantly expedite morphology transformation process of PKK‐S‐PEG + NIR group by approximately 2–3 times. Strikingly, in addition to fast morphological change, the intense PA signal at tumor site (Figure 7E) revealed that this strategy also distinctly promoted the accumulation of PKK‐S‐PEG. Thus, PAI provides an effective strategy for quantitatively monitoring the transformation process of peptides in vivo, which provides valuable view of in vivo self‐assembly.

### 2.4 Radionuclide labeling for quantitative evaluation in vivo fate

Radionuclide labeling (RL) is a commonly‐used tool for tracking molecules of interest in vivo.^[^ ^103^ ^]^ Rays (γ‐ray) emitted from radionuclides are highly penetrating and sensitive. Positron emission tomography (PET) and single photon emission computed tomography (SPECT) serve as routine imaging tools to detect radiotracers. Currently, ^125^I, ^68^Ga‐, and ^18^F are commonly employed to label molecules or nanostructures for tracking.^[^ ^103^ ^]^ Meanwhile, the rays emitted from radioactive isotope are quantifiable as their intensity is positively related to the content of isotope. Consequently, by introducing isotope, RL‐based quantitative analysis facilitates accurately probing in vivo fate of self‐assembled peptides, including blood circulation time, biodistribution, and target tissue retention.

**FIGURE 8:**
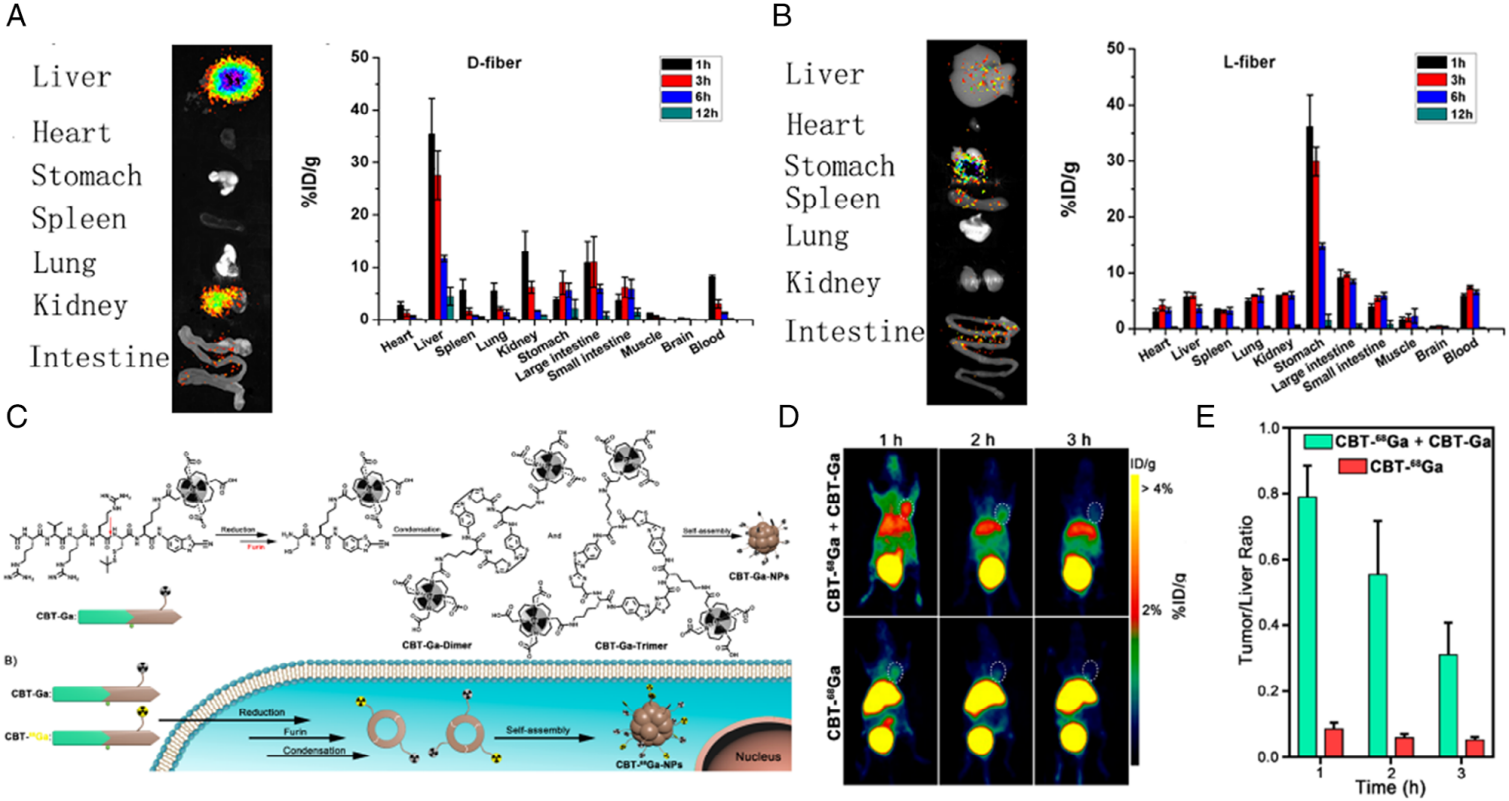
Radionuclide labeling for the detection of biodistribution of self‐assembled peptides. Ex vivo images and quantitative analysis of major organs of mice respectively treated with ^125^I‐labelled d‐peptide (A) and l‐peptides (B) at 1 h of post‐injection. Reproduced with permission.^[^ ^29^ ^]^ Copyright 2015 American Chemical Society. (C) Molecule design of peptide‐based furin‐responsive radiotracer and schematic of furin‐instructed self‐assembly into ^68^Ga nanoparticles. (D) Coronal microPET images of xenograft tumor mice at 1, 2 and 3 h upon injection of CBT‐^68^Ga and CBT‐Ga (top) or CBT‐^68^Ga (bottom). White dashed ellipses indicate the site of the tumor. (E) The tumor/liver ratios by PET quantification at 1, 2, and 3 h of post‐injection (% ID g^−1^, *n* = 4 for each group). Reproduced with permission.^[^ ^106^ ^]^ Copyright 2019 American Chemical Society.

Tracking biodistribution in vivo contributes to revealing metabolic process and biosafety of peptide, which lays the foundation for clinical translation of self‐assembled peptides.^[^ ^104^ ^]^ Yang and co‐workers studied the biodistributions of l‐ and d‐peptide‐based nanofibers.^[^ ^29^ ^]^^125^I‐radiolabeling on peptides can be achieved on the tyrosine residue using chloramine‐T method. This study unraveled that l‐fibers preferentially accumulated in digestive system, especially the stomach. d‐fibers primarily distribute in liver and kidney (Figure 8A,B). Both of them showed no accumulation in the thyroids within 12 h upon administration. These results could be attributed to the stability and existing state of l‐ and d‐ peptides. Due to the inadequate biostability of l‐peptide, it was degraded and existed in free state in vivo, which leaded to the similar distribution to free ^125^I, accumulating in gastrointestinal tract.^[^ ^105^ ^]^ Conversely, better stability of d‐peptide aided in maintaining nanostructures in vivo. Thus, majority of d‐peptide‐based nanofibers were captured by reticuloendothelial system (RES), resulting in distributing in the liver and kidney within 3 h. Additionally, radioactive signals of both l‐ and d‐fibers cannot be detected at 12 h post injection, corroborating that nanofibers were quickly eliminated from the body, without cumulative toxicity. These results elucidated the in vivo distribution and metabolic characteristics of l/d‐peptide‐based nanomaterials, laying foundation for further biomedical application.

Apart from biodistribution, accumulation at the site of interest is also an important aspect of the study of self‐assembled peptides. Liang and coworkers utilized peptide and radionuclide ^68^Ga to construct a furin‐responsive radiotracer (CBT‐^68^Ga).^[^ ^106^ ^]^ The cleavage of CBT‐^68^Ga by trans‐Golgi protease furin triggered a click condensation reaction, after which the residues self‐assembled into nanoparticles (Figure 8C). Co‐administration of CBT‐^68^Ga with its non‐radioactive analogue CBT‐Ga could prompt the formation of ^68^Ga nanoparticles (CBT‐^68^Ga‐NPs) in furin‐overexpressed tumor cells, significantly enhancing microPET signals in vivo, which could quantitatively probe the retention of material. The use of CBT‐Ga secured the self‐assembly of CBT‐^68^Ga‐NPs by eliminating the interference of intercellular free cysteine. As shown in Figure 8D, in vivo experiments corroborated that signals in the tumor tissues were clearly detected by microPET at 1 h after intravenous co‐injection of CBT‐^68^Ga and CBT‐Ga. Conversely, after only injection of CBT‐^68^Ga, radiotracer accumulated much more in the liver than in the tumor. This indicated that the combination of CBT‐^68^Ga and CBT‐Ga induced the formation of CBT‐^68^Ga‐NPs in tumors. Of note, the mean tumor/liver ratio of the experimental tumors is approximately 9.1 folds as high as that of the control tumors by quantitatively analyzing radioactive signals of organs and tumors at 1 h post injection (Figure 8E), indicative of increased retention in tumor. This study demonstrated that RL‐based quantification produces comparable data to assess accumulation of nanostructures in targeting region.

In addition, in order to investigate the in vivo circulation and biodistribution profiles of self‐assembled peptides, Xu's group labelled them with radioactive ^125^I.^[^ ^46^ ^]^ After intravenous injection of different cholesterol derivatives labeled with ^125^I, radioactive signals were mainly observed in the liver, followed by blood, spleen, lung and other organs. Compared with foregoing Yang's work,^[^ ^29^ ^]^ cholesterol‐peptide conjugates had longer blood circulation than other self‐assembled peptides. Overall, RL is a facile and sensitive tool for providing accurate information of in vivo fate of self‐assembled peptides.

### 2.5 Computed tomography for probing self‐assembly

**FIGURE 9:**
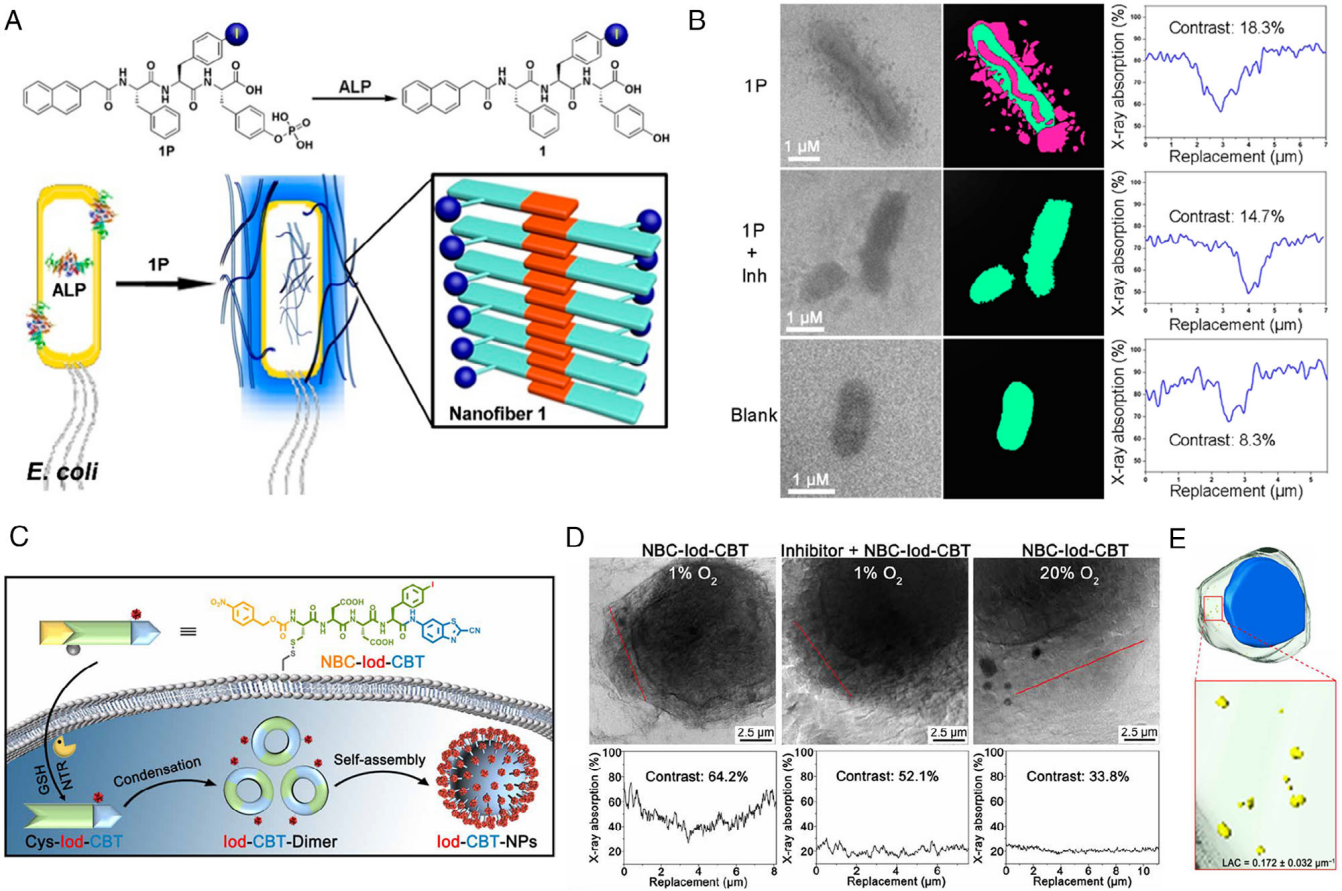
Computed tomography for probing self‐assembly (A) Molecule structure of 1P and formation of nanofibers from 1P catalyzed by bacterial ALP. (B) Soft X‐ray images (left), segmentation (middle), and absolute soft X‐ray absorptions (right) of *E. coli* treated with 1P, 1P together with ALP inhibitor and blank *E. coli* without treatment. Reproduced with permission.^[^ ^48^ ^]^ Copyright 2016 American Chemical Society. (C) Illustration of NTR‐induced self‐assembly of NBC‐Iod‐CBT into Iod‐NPs inside a hypoxic cell. (D) 2D projection images (top) and cytoplasm CT contrast (bottom) of hypoxic HeLa cells treated with NBC‐Iod‐CBT under different conditions. (E) Reconstructed 3D image and magnified view of hypoxic HeLa cell treated with NBC‐Iod‐CBT. Reproduced under the terms of CC BY‐NC license.^[^ ^47^ ^]^ Copyright 2022, Zhu et al.

Computed tomography (CT) is classical imaging technique using X‐ray or gamma ray to produce high‐resolution cross‐sectional images of internal body.^[^ ^107^ ^]^ These cross‐sectional images are collected and stacked by computers to form a 3D image, so as to discriminate the basic structures and lesions. However, CT generally encounters low sensitivity for molecular imaging. Thus, heavy atoms such as iodine‐, tungsten‐, and barium‐based contrast agents are employed to augment CT signals due to their ability to increase CT attenuation.^[^ ^107^ ^]^ Recently, nano‐computed tomography (nano‐CT) is developed to observe self‐assembly at the cellular level.^[^ ^108^ ^]^ Nano‐CT is characterized as a high‐resolution and cross‐sectional imaging technique. Benefiting from a transmission target X‐ray tube, the diameters of focal spot can be downsized to less than 400 nm. High spatial resolution will be achieved by specific detectors, far beyond the resolution of micro‐CT systems, enabling quantitative characterization of intracellular structures. In the study of Liang's group, an iodine‐bearing self‐assembled peptide, Nap‐Phe‐Phe(I)‐Tyr(H_2_PO_3_)‐OH (1P), was synthesized and applied to nano‐CT imaging of ALP activity in bacteria (Figure 9A).^[^ ^48^ ^]^ 1P self‐assembled into nanofibers after dephosphorylation by ALP‐overexpressed *E. coli*. Self‐assembly would concentrate the heavy atom iodine, enhancing the CT contrast of ALP location. The results (Figure 9B) showed that formed nano‐assemblies could be observed inside and on the membrane of *E. coli*, and the nano CT imaging revealed that ALP‐overexpressed *E. coli* had a CT contrast of 18.3%, whereas the blank cells only possessed a CT contrast of 8.3%.

In addition, Liang's group also designed an iodine‐containing peptide precursor strategy, which subjected to nitroreductase (NTR) cleavage and CBT‐Cys click condensation reaction to form nanoparticles in hypoxia microenvironment.^[^ ^47^ ^]^ As shown in Figure 9C, the peptide molecule (NBC‐Iod‐CBT) was consist of a nitroreductase cleavage substrate (4‐nitrobenzyl carbamate, NBC), a latent cysteine moiety and 2‐cyano‐benzothiazole (CBT) for click reaction, an iodinated phenylalanine for CT imaging, and two aspartic acid for good water solubility. Upon the disulfide bond was reduced by GSH and the NBC substrate is cleaved by NTR, the CBT‐Cys click reaction happened immediately between two NBC‐Iod‐CBT molecules, yielding cyclic dimer Iod‐CBT‐Dimer. Iod‐CBT‐Dimer would assemble into nanoparticles Iod‐CBT‐NPs. The 2D projection images and quantitative results of three groups of HeLa cells treated with NBC‐Iod‐CBT under different conditions (Figure 9D) showed that hypoxic cells had the highest cytoplasm CT contrast of 64.2%. Hypoxic cells with NTR inhibitor and normoxic cells possessed relatively low cytoplasm CT contrast of 52.1% and 33.8% respectively, suggesting the enhanced CT contrast was achieved by hypoxia‐induced NTR overexpression. Since each substance exhibits differentiated absorption capacity, the formation of nanostructures could be distinguished according to the different values of linear absorption coefficient (LAC) derived from nano‐CT. By using nano‐CT, the average LAC value of the nanoparticles in the 3D rendering image was determined to be 0.182 ± 0.078 μm^−1^ in vitro, while the average LAC value of the background was calculated to be 0.016 ± 2.043 × 10^−4^ μm^−1^. In the hypoxia HeLa cells (Figure 9E), the average LAC value in cytoplasm was calculated to be 0.172 ± 0.032 μm^−1^, which was close to that of formed nanoparticles in vitro. However, in the normoxic cells, the average LAC values were much lower. This result indicated that the nanoparticles were formed in hypoxic HeLa cells. Based on the quantifiable data, this strategy could serve to precisely monitor the intracellular formation of nanostructures.

### 2.6 Multimodal imaging for quantitatively detecting biomarkers

To date, simultaneously achieving high spatial resolution, sufficient sensitivity, and tissue penetration by a single technique remains a daunting task.^[^ ^109^ ^]^ Meanwhile, since some regions of interest feature deep location and small volume, correct and accurate detection only based on one technique is considerably challenging. Therefore, bimodal and multimodal imaging which use two or more imaging techniques to visualize and quantify the same subject are considered as feasible strategies to overcome the limitations.^[^ ^110^ ^]^ Benefiting from the integrating advantages of each technique, various information with high spatiotemporal resolution and sensitivity can be provided. Self‐assembled peptide with multiple modification sites is an ideal nanocarrier to accommodate multimodal imaging agents.^[^ ^111^ ^]^ Taking advantage of enzyme catalysis and ligand‐receptor interaction, self‐assembled peptides can switch on multimodal imaging signals, allowing for the quantitative detection of enzyme expression or bacterial infection, which facilitates the diagnosis and treatment of diseases.

**FIGURE 10:**
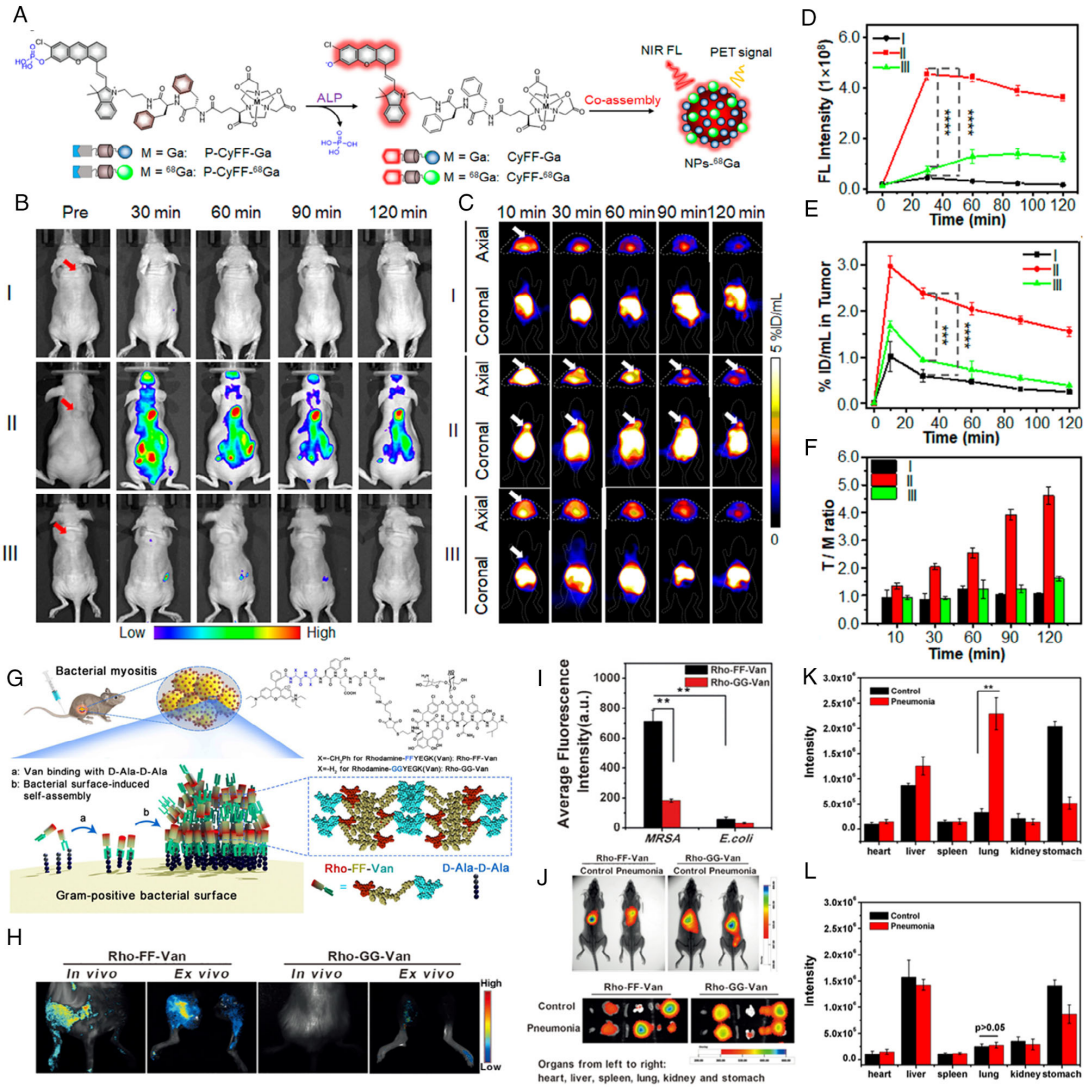
Multimodal imaging for quantitatively detecting biomarkers. (A) Molecular structure of P‐CyFF‐^68^Ga and Schematic of ALP‐instructed co‐assembly for FL and PET bimodal imaging. (B) FLI and (D) FL signal intensity of tumor‐bearing mice with various treatment at different time points. (C) Axial and coronal PET imaging and (E) of tumor‐bearing mice with various treatment at different time points. (F) The ratio of tumor to muscle (T/M) at different time points. The I is for injection of P‐CyFF‐^68^Ga; the II is for injection of P‐CyFF‐^68^Ga and P‐CyFF‐Ga; the III is for pre‐injection of Na_3_VO_4_ for 30 min and injection of P‐CyFF‐^68^Ga and P‐CyFF‐Ga. Reproduced with permission.^[^ ^112^ ^]^ Copyright 2019 American Chemical Society. (G) Molecular structure of Rho‐FF‐Van and Rho‐GG‐Van and schematic of self‐assembly of Rho‐FF‐Van on the surface for in vivo gram‐positive bacterial infection imaging. (H) FL images (top) of in vivo and ex vivo MRSA‐infected myositis left hindlimb and *E. coli*‐infected myositis right hindlimb at 2 h after intravenous injection of Rho‐FF‐Van or Rho‐GG‐Van. (I) Quantification of average FL intensity of ex vivo tissues. (J) In vivo images (top, overlay of X‐ray and isotope signals) of MRSA‐induced pneumonia mice; ex vivo images (bottom) of organs at 0.5 h after intravenous injection of ^125^I‐Rho‐FF‐Van or ^125^I‐Rho‐GG‐Van. (K) In vivo distribution of ^125^I‐Rho‐FF‐Van in control and pneumonia mice. (l) In vivo distribution of ^125^I‐Rho‐GG‐Van in control and pneumonia mice. Reproduced under the terms of CC BY‐NC license.^[^ ^26^ ^]^ Copyright 2017, Yang et al.

For multimodal imaging induced by enzyme catalysis, Ye's group designed an activable FLI/PET bimodal nanoprobe (P‐CyFF‐^68^Gd) and its cold analogue (P‐CyFF‐Gd) for bimodal imaging of ALP enzyme activity.^[^ ^112^ ^]^ As shown in Figure 10A, overexpressed enzyme, alkaline phosphatase (ALP), on the cell membrane was employed to activate the fluorescence by dephosphorylation, after which P‐CyFF‐^68^Gd and P‐CyFF‐Gd in situ co‐assembled into nanoparticles, simultaneously switching on PET signals. In vivo bimodal imaging results showed that after intravenous injection of P‐CyFF‐^68^Gd and P‐CyFF‐Gd, fluorescence (Figure 10B,D) in the tumor region reached the highest level at 30 min, which was approximately 6.2 folds as high as that pretreated with ALP inhibitor (Na_3_VO_4_). Akin to FLI, PET imaging (Figure 10C) quickly displayed signals at 30 min in tumors upon injection of P‐CyFF‐^68^Gd and P‐CyFF‐Gd. Quantification (Figure 10E) results show that about 2.4% ID mL^−1^ of ^68^Ga were accumulated in the tumor treated with P‐CyFF‐^68^Gd and P‐CyFF‐Gd. Moreover, the ratio of tumor to muscle (T/M) gradually ascended from 1.3 at 10 min to 4.6 at 2 h (Figure 10F), indicating that the tumor issue could be discriminated from the surrounding tissues by axial and coronal PET imaging. These studies corroborated that concomitant NIR FL and PET imaging of co‐assembly of P‐CyFF‐^68^Gd and P‐CyFF‐Gd are capable of efficiently detection of enzyme activity in vivo.^[^ ^112^ ^]^ Based on the similar molecular structure, Ye and co‐workers further developed a trimodal imaging probe.^[^ ^113^ ^]^ A trans‐cyclooctene (TCO) was introduced to form P‐FFGd‐TCO. P‐FFGd‐TCO was capable of in situ self‐assembling into nanoaggregates (FMNPs‐TCO) on the tumor cell membrane in the response to ALP upon i.v. injection, generating amplified near‐infrared fluorescence and MRI signals. Subsequently, a radioisotope labeled Tz (Tz‐^68^Gd) was administrated intravenously, and it would couple with pre‐targeted FMNPs‐TCO via the bioorthogonal reaction, enabling radioactivity signals for PET imaging. The results showed that, through pre‐targeted strategy and bioorthogonal reaction, the quantification of FLI, MRI and PET signals could precisely reflect the ALP activity in tumor.

Additionally, in the study of Gao's group, a MMP‐2‐activatable peptide‐based probe was fabricated for fluorescence/photoacoustic dual‐modal imaging.^[^ ^114^ ^]^ This probe was composed of a near‐infrared dye (Cy5.5), a peptide substrate of MMP‐2 and a quencher (QSY21). The probe displayed MMP‐2 concentration dependent fluorescence after MMP‐2 cleavage. More importantly, the photoacoustic signals at 680 nm could be used to quantitatively detect the level of MMP‐2 expression in breast cancer in vivo.

Based on ligand‐receptor interaction, Yang's group developed a dual‐modal fluorescent and radionuclide probe to simultaneously achieve the superficial and deep infection imaging (Figure 10G).^[^ ^26^ ^]^ Vancomycin and rhodamine were combined with phenylalanine‐phenylalanine (FF) to form peptide derivatives (Rho‐FF‐Van), which could aggregate on the surface of methicillin‐resistant *S. aureus* (MRSA) by the interaction between Vancomycin and d‐Ala‐d‐Ala. The probe showed stronger fluorescence signals in muscular MRSA‐infected mice than *E. coli*‐infected mice (Figure 10H). The quantification of fluorescence intensity illustrated that Rho‐FF‐Van generated 3.9 times the fluorescence of non‐assembled Rho‐GG‐Van in the MRSA‐infected mice (Figure 10I), resulting from the assembly‐enhanced AIE effects. Notably, Rho‐FF‐Van selectively generated intense fluorescence in the MRSA‐infected tissue, which was approximately 8.7 times as high as in the *E. coli*‐infected tissue. Moreover, Rho‐FF‐Van was labeled by iodine‐125 on tyrosine, forming ^125^I‐Rho‐FF‐Van for deep‐tissue infection imaging. In the pneumonia model, the ^125^I‐Rho‐FF‐Van probe significantly increased the radioactive signal in the thoracic cavity (Figure 10J). Quantitative analysis indicated that radioactive signals from the pneumonia mice treated ^125^I‐Rho‐FF‐Van were approximately 8.9‒13.3 times as high as pneumonia mice treated with non‐assembled ^125^I‐Rho‐GG‐Van or control mice treated with ^125^I‐Rho‐FF‐Van or ^125^I‐Rho‐GG‐Van, respectively (Figure 10K,L). Biodistribution analyzed by ex vivo organ radioactive imaging further verified the distribution in lung of ^125^I‐Rho‐FF‐Van. Consequently, quantitative analysis of Rho‐FF‐Van and ^125^I‐Rho‐FF‐Van verifies the capacity for detection of superficial and deep bacterial infection in vivo.

### 2.7 Cryo‐Electron microscopy for detecting nanostructures of self‐assembled peptides with high‐resolution

**FIGURE 11:**
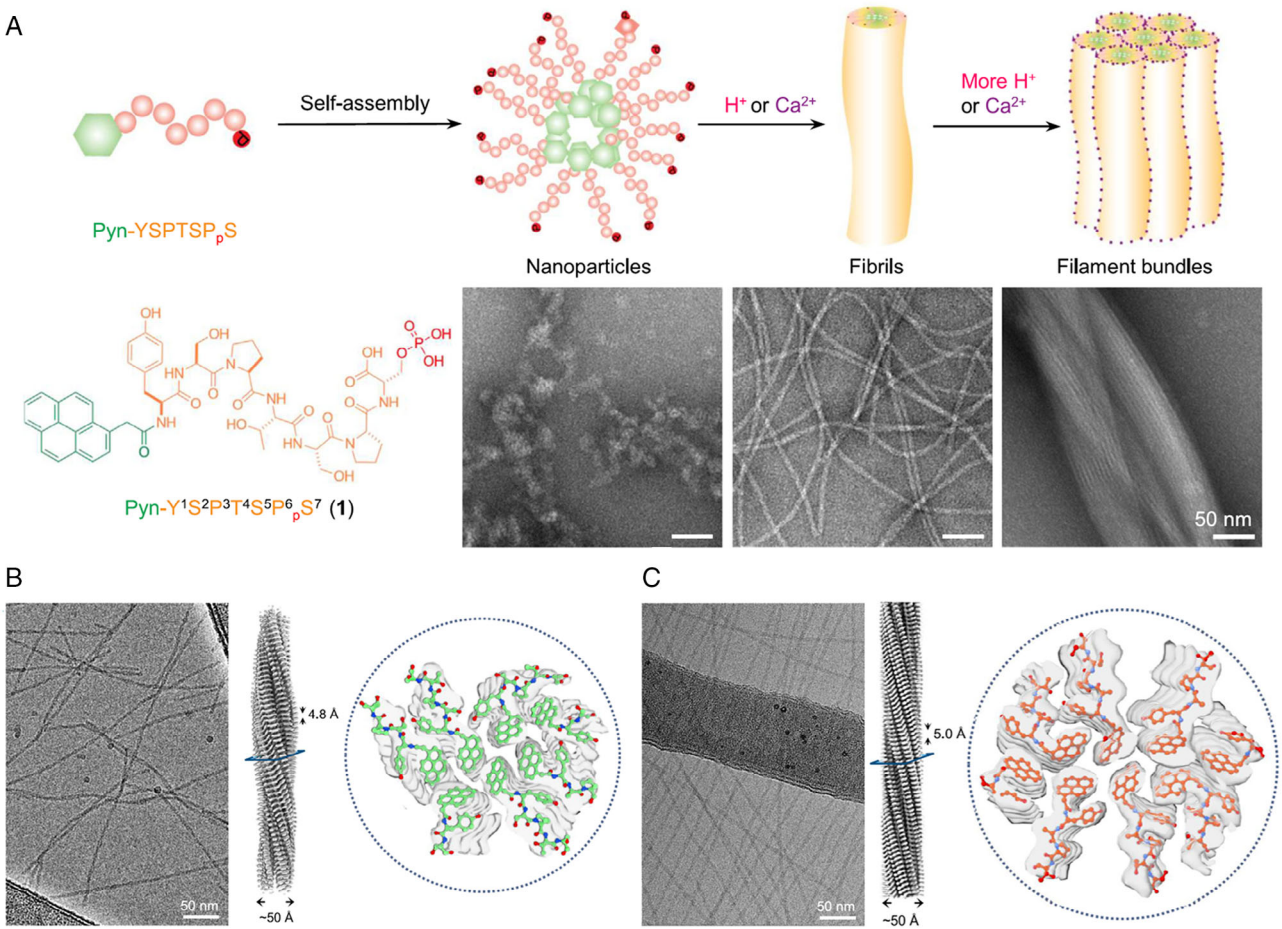
Cryo‐EM for detecting nanostructures of self‐assembled peptides with high‐resolution. (A) Illustration of the hierarchical assembly of an IDP and the corresponding assemblies at three stages. (B) Cryo‐EM images (left), 3D helical reconstruction (middle), and cross‐section view (right) of the filaments of 1 (pH = 2.0). (C) Cryo‐EM images (left), 3D helical reconstruction (middle), and cross‐section view (right) of the filaments of 1 (pH = 4.0, 2.0 mm Ca^2+^). Reproduced with permission.^[^ ^52^ ^]^ Copyright 2023 Elsevier.

Electron microscopy (EM) is among important techniques to characterize the morphology and structure of self‐assembled peptides. However, the structure and morphology of samples with high water contents are invariably changed or damaged by conventional sample preparation procedures, resulting in distortions and misinterpretations of nanostructures.^[^ ^52^ ^]^ The invention of Cryo‐electron microscopy (Cryo‐EM) has overcome this defect perfectly. By rapidly freezing the self‐assembled peptides at low temperatures, liquid water was instantaneously transformed into amorphous ice, ensuring high vacuum and reducing electron radiation damage,^[^ ^115^ ^]^ which allows for observation of self‐assembled peptides in the native state with high resolution. Furthermore, the three‐dimensional reconstruction is introduced to describe the structural details of peptide assemblies.^[^ ^51^ ^]^ In the study of Xu's group, cryo‐EM was used to interpret the hierarchical assembly of intrinsically disordered short peptides (IDPs).^[^ ^52^ ^]^ An IDP was constructed based on YSPTSPS by conjugating an aromatic motif to the N‐terminus and introducing a phosphate group on serine. Under the acidic environment or adding calcium ions, the IDP transformed from nanoparticles to nanofibers (Figure 11A). With the cryo‐EM, the nanofiber of IDP was illustrated to be C1 symmetry with a helical rise of 4.74 Å and a twist of 5.38° at pH 2 (Figure 11B). At the condition of pH 4 and 2.0 mm Ca^2+^, the nanofiber was C2 point group symmetry with 4.95 Å helical rise and −3.80° helical twist (Figure 11C). Given that fibrillar structures are common peptide assemblies, cryo‐EM structural reconstruction constitutes a potent method for providing atomistic structures and data of peptide assemblies.

## 3 LIQUID CHROMATOGRAPHY‐MASS SPECTROMETRY FOR QUANTITATIVE ANALYSIS OF SELF‐ASSEMBLED PEPTIDES

Liquid chromatography‐mass spectrometry (LC‐MS) is an accurately qualitative analysis technique, due to the peak areas are directly proportional the content of the tested substance.^[^ ^116^ ^]^ LC‐MS, which utilizes liquid chromatography as the separation system and mass spectrometry as the detection system, is characterized by fast analysis, high separation, high sensitivity, and ability to provide information of molecule weights and chemical structures. Benefiting from these merits, LC‐MS is widely used in various fields including drug development,^[^ ^117^ ^]^ pharmacokinetics,^[^ ^118^ ^]^ and clinical diagnosis.^[^ ^119^ ^]^ Since it provides structural and quantitative data, LC‐MS can be applied to the research of stability^[^ ^120^ ^]^ and endocytosis,^[^ ^121^ ^]^ which constitutes an important process before the clinical translation.

Yang and coworkers studied the effects of amino acid configuration on biostability of peptides by LC‐MS.^[^ ^29^ ^]^ In this study, the d‐peptide was incubated with plasma in vitro for different hours, to simulate the internal environment after injection. The LC‐MS data showed that the residues of the Nap‐G^D^F^D^F^D^YGRGD (d‐fiber) were much more than those of Nap‐GFFYGRGD (l‐fiber) in plasma upon incubation for 24 h, suggesting that the biostability of peptide‐based nanomaterials can be improved by employing the d‐amino acid. Additionally, Liu's group used LC‐MS to investigate if the glucose modification could increase biostability of self‐assembled peptides.^[^ ^53^ ^]^ The results of LC‐MS showed that after 24 h of incubation with proteinase K, glucose‐modified peptide was less digested than peptide without glucose modification did, corroborating that glucose modification is an effective approach for improving the biostability of self‐assembled peptides.

Secondary structures also play an indispensable role in stability and cellular uptake of self‐assembled peptides. Liang and co‐workers added protease K to the α‐helix nanofibers and β‐fold conformation nanoparticles respectively.^[^ ^18^ ^]^ After incubation with protease for 24 h, the residue of peptides in nanofibers was more than that in nanoparticles. Subsequently, HeLa cells were employed for cellular uptake. The intracellular peptide concentration of nanofibers group was higher than nanoparticles group at every time point, as evidenced by the quantitative analysis of LC‐MS. Accordingly, nanofibers exhibited better stability against enzyme digestion and better cellular uptake than nanoparticles, which provides valuable evidences and guides for designing self‐assembled peptides.

## 4 MOLECULAR DYNAMICS SIMULATION FOR QUANTITATIVE ANALYSIS OF SELF‐ASSEMBLY

Self‐assembly and folding of peptides constitutes a multi‐scale process ranging from atomic and molecular level to mesoscopic level,^[^ ^122^ ^]^ eventually leading to delicate nanostructures or disorderly aggregates.^[^ ^123^ ^]^ A variety of variables engage in the constructions of self‐assembly, including molecular conformational preference,^[^ ^124^ ^]^ intermolecular interactions,^[^ ^125^ ^]^ and interactions between peptides and their surroundings (e.g. solvents, ions), rendering the mechanism of peptide self‐assembly elusive.^[^ ^2^ ^]^ Thorough understanding the microscopic behaviors of self‐assembly and structure‐to‐function relationships only by conventional experimental methods is a strenuous and demanding task. Recently, molecular dynamics (MD) simulation has increasingly become a powerful characterization method for investigating biological molecules in atomic resolution.^[^ ^126^, ^127^ ^]^ Since the first MD simulation in the 1970s,^[^ ^128^ ^]^ advances in computer technologies harbor the iterations of MD simulation program. Complex molecular trajectories during self‐assembly can be clearly calculated and reproduced in silico.^[^ ^41^, ^129^ ^]^ Quantifiable information related to self‐assembly, such as the molecular conformation and intermolecular interactions, can be calculated based on dynamics parameters gathered from MD simulation program, describing the nanostructures in detail, which has not been available experimentally.^[^ ^130^ ^]^ Although the accuracy of MD simulation is required to be verified through experiments, simulated microscopic interactions and events could provide valuable insights into mechanism of self‐assembly from multi‐scale perspectives. Since a wide variety of functions can be achieved by MD simulation, we propose several examples in the following section to illustrate its potential in studying the relationship between structure and microscopic morphology of self‐assembled peptides.

### 4.1 MD simulation for quantifying the content of secondary structures

**FIGURE 12:**
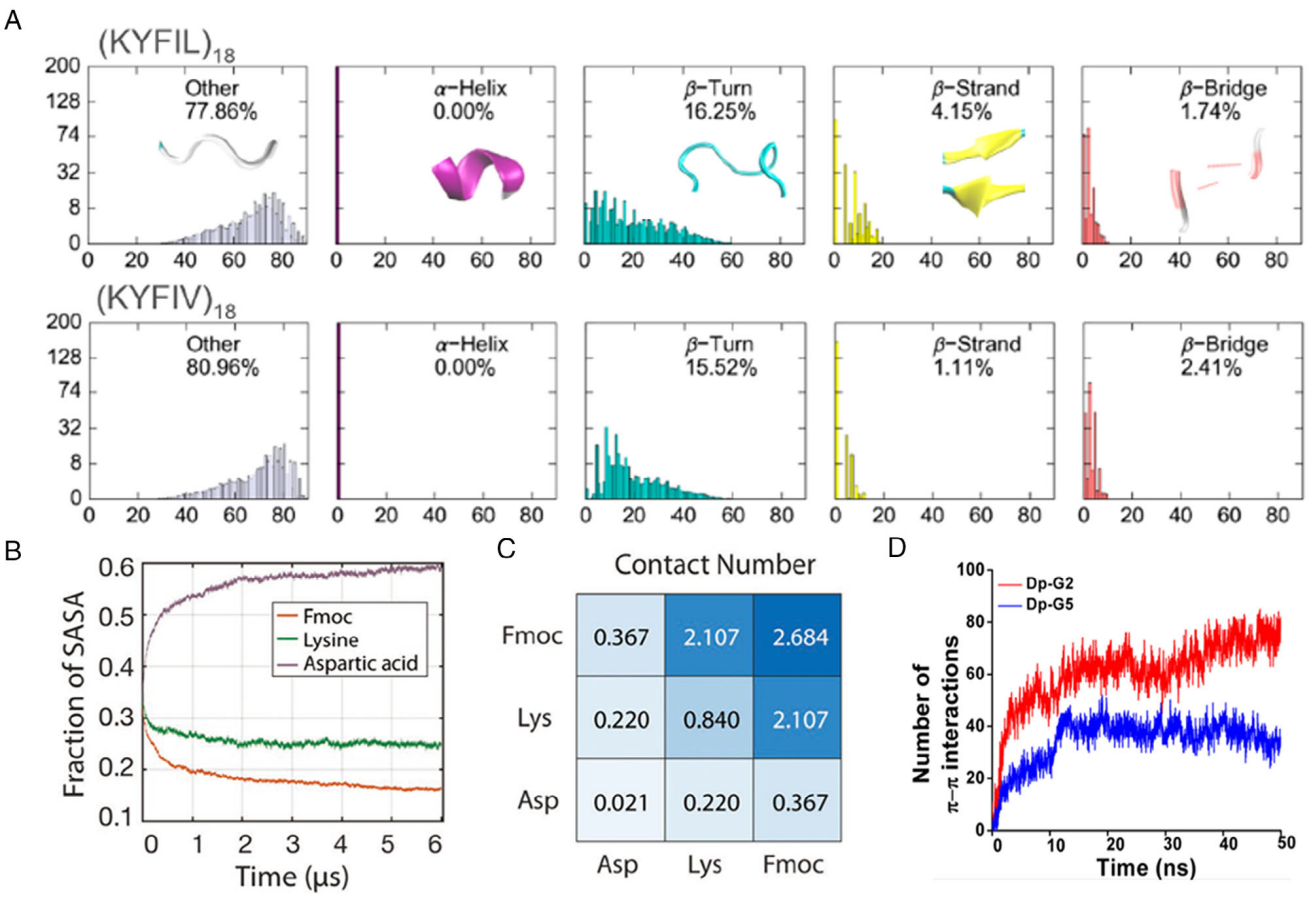
Molecular dynamics simulation for the calculation of key parameters during self‐assembly. (A) Proportions of pentapeptides (KYFIL and KYFIV) secondary structures. Reproduced with permission.^[^ ^42^ ^]^ Copyright 2019 American Chemical Society. (B) SASA fraction of the Fmoc, lysine, and aspartic acid groups at different simulation time. (C) Contact number for different pair of groups at 1μs of simulation. Reproduced under the terms of CC BY‐NC license.^[^ ^41^ ^]^ Copyright 2021, Chakraborty et al. (D) The number of π─π interactions in Dp‐G2 and Dp‐G5 within simulation. Reproduced with permission.^[^ ^33^ ^]^ Copyright 2022 American Chemical Society.

It has been proved that secondary structures could dictate assembly morphologies and physicochemical properties of self‐assembled peptides.^[^ ^2^ ^]^ Molecular conformation of peptide could be qualitatively detected by circular dichroism (CD) and Fourier transform infrared (FTIR). However, a peptide chain may simultaneously contain several different secondary structures. Currently, the content of secondary structures can be approximately estimated by computational approaches. Tang et al. leveraged MD simulation to characterize conformational propensity of pentapeptides (Figure 12A).^[^ ^42^ ^]^ The secondary structural content of all possible conformations was quantitatively calculated, corroborating gelling peptide (KYFIL) contained more proportion of β‐strand (4.15%) than non‐gelling peptide (KYFIV) did (1.11%). Additionally, Xu et al. used DichroWeb program to estimate the fraction of secondary structure of enzyme‐instructed peptide assemblies based on the CD spectrum.^[^ ^131^ ^]^ The results indicated that the hydrogel primarily displayed β‐sheet conformation (50%), followed by unordered structure (25%), turn (23%) and α‐helix (2%). Therefore, by approximately quantifying the content of secondary structures, it has been proved that β‐strand or β‐sheet plays a dominant role in formation of ordered peptide assemblies.

### 4.2 MD simulation for parameter‐based analysis of intermolecular alignment

Recording and interpreting of dynamics parameters within MD simulation is a useful approach for investigating the process of self‐assembly. In this regard, solvent accessible surface area (SASA) and contact numbers of different groups act as important data for unraveling the intermolecular interactions within the process of nanofiber formation.^[^ ^132^ ^]^ Chakraborty et al. designed an hydrogelator (Fmoc‐Lys(Fmoc)‐Asp) with fairly low critical gelation concentration.^[^ ^41^ ^]^ SASA and contact numbers were calculated to study the roles and interactions of the Fmoc, Asp, and Lys groups within self‐assembly. As shown in Figure 12B,C, the decreasing SASA fraction of Fmoc and Lys and high contact number of Fmoc/Fmoc and Fmoc/Lys indicated that Fmoc group highly interacted with each other and with Lys group, and these two groups tended to buried inside hydrophobic core of nanofibers. Therefore, the dynamics parameters can estimate microscopic events within self‐assembly.

### 4.3 MD simulation for determining the number of non‐covalent interactions within self‐assembly

To investigate the effect of molecular structure on luminescence efficiency, Zhang and co‐workers synthesized a series of AIE‐conjugated peptides by adjusting the number of glycines between AIEgen (TPE) and self‐assembled sequence (FFF).^[^ ^33^ ^]^ Fluorescence spectra indicated that fluorescence intensity of peptide containing two glycines (Dp‐G2) was the strongest, while fluorescence intensity of peptide containing five glycines (Dp‐G5) was the weakest. In order to study the structure‐function relationship, the number of non‐covalent interactions during self‐assembly were calculated by MD simulation. The results (Figure 12D) showed that the number of intermolecular hydrophobic interactions and π─π stacking of Dp‐G2 was approximately 2.27 times and 2.39 times more than those of Dp‐G5. This suggested that the freedom and motion of TPE reduced as it located near the self‐assembled sequence. Consequently, assembly is capable of restricting the intramolecular motions of AIEgens when the length of linker was less than 5 glycines, leading to stronger fluorescence.

## 5 CONCLUSION AND OUTLOOK

In this review, the techniques for quantitative analysis of self‐assembled peptide were described in detail, including imaging‐based quantitative, LC‐MS and molecular dynamics simulation. Currently, advances in detecting technology enable researchers to conduct multifaceted characterization on structures and properties of self‐assembled peptides at macro and micro levels, so as to guide the design of novel functional nanomaterials. Although qualitative investigation of self‐assembled peptides, such as characterizing conformation by CD spectrum, could illustrate profiles of self‐assembled peptides to some extent, quantitative analysis can provide more comprehensive and detailed information, achieving a better research gain. Of note, quantification of in vivo self‐assembled peptides behaviors (e.g. aggregation degree and rate of transformation) or in silico simulation of self‐assembly‐relevant parameters (e.g. SASA and contact number) would elucidate the underlying mechanism of supramolecular self‐assembly. Besides, detection of in situ concentration of self‐assembled peptides can achieve many desired functionalities, including detecting biomarkers and guiding precise therapy. Evolution of quantitative analysis techniques for self‐assembled peptides is considerably conducive to providing prized knowledge of supramolecular self‐assembly and promoting therapeutic applications. Benefiting from the excellent biocompatibility and low immunogenicity, functional peptides are promising in the diagnosis and treatment of diseases, for example some peptide‐drug conjugates (PDC) have been approved for marketing^[^ ^133^ ^]^ and radionuclide‐labeled peptide probes have advanced into the stage of clinical trials.^[^ ^134^ ^]^ Although the quantitative analysis of self‐assembled peptides remains in the laboratory stage, determination of self‐assembly mechanism, biostability, biodistribution, and tissue retention allows researchers to understand the in vivo fate of self‐assembled peptides, which plays an essential role in the process of clinical translation.

However, there are still challenges to be conquered in this field.

1. In vivo degradation of peptide would compromise the accuracy of imaging‐based analysis methods, such as “always on” fluorophores‐based FLI and radionuclide labeling, because the separation of signal‐emitting groups from the peptide molecules would lead to intense interfering signals in the untargeted region. In this regard, more advantageous fluorophores (e.g. NBD and AIEgens) whose luminescence depends on the formation of nanostructures and more stable radionuclide labeling method should be explored to circumvent this defect.
2. Although high‐penetration PAI show great potential in guiding precise therapy and detecting in situ self‐assembly, the resulting images are still two‐dimension, resulting in limited information. AI algorithm and software should be developed to integrate 2D images to reconstruct 3D images, better illustrating the distribution in the target tissue.
3. Self‐assembled peptides used for CT have rarely been exploited so far, suggesting that some combinations between self‐assembled peptides and imaging groups remain unexplored.
4. For analysis of in vivo and cellular samples, LC‐MS often suffers limited sample throughout. Meanwhile, since cells were atomized and ionized within the analysis, LC‐MS cannot be used for dynamic detection. Thus, more advanced instrument, such as mass cytometry, are required for higher throughout, greater efficiency, and for accurately dynamic detection of the molecule changes in vivo and intracellularly, substitute quantitative method with similar capacity should be proposed.
5. To date, most quantitative analysis of self‐assembled peptide concentration, behaviors and biological functions are performed at the cellular or higher level. The in situ, dynamic and high‐resolution monitoring at subcellular level remains a challenge due to the technological limitations. Therefore, considerable and integrated efforts should be devoted to the design and development of organelle‐based and high‐resolution techniques for accurate sensing self‐assembled peptides intracellular behaviors and interactions with intracellular structures.
6. Although MD simulation is a powerful tool for expediting exploration of peptide self‐assembly system, inconsistent results may appear compared with actual experiments, due to the simplification of the force field. Thus, it is imperative to improve existing force field and develop new substitutes for increasing accuracy and efficiency, which exerts maximal efficacy of MD simulation in the investigation and design of novel peptide‐based nanomaterials.
7. Finally, complicated intracellular and in vivo environments have a negative impact on accuracy of imaging‐based quantitative techniques, resulting from interfering biological macromolecules and signal‐absorbing tissues. No single imaging‐based method, thus far, is able to simultaneously provide overall information with high sensitivity and spatial resolution. Therefore, in the future, development of integrated techniques with cooperative efforts of researchers from biomedicine, chemistry, and physics would aid in solving these issues.

We hope that, with this review, greater endeavors will be made to extend application of current techniques and explore novel tools for more efficiently quantitative analysis of self‐assembled peptides.

## CONFLICT OF INTEREST STATEMENT

The authors declare no conflicts of interest.
